# Proline-based organocatalyst-mediated asymmetric aldol reaction of acetone with substituted aromatic aldehydes: an experimental and theoretical study

**DOI:** 10.3906/kim-1908-3

**Published:** 2020-04-01

**Authors:** Nevin ARSLAN, Selami ERCAN, Necmettin PİRİNÇÇİOĞLU

**Affiliations:** 1 Department of Chemistry, Faculty of Science, Dicle University, Diyarbakır Turkey; 2 Department of Field Crops, Faculty of Agriculture, Şırnak University, İdil, Şırnak Turkey; 3 Department of Nursing, School of Health, Batman University, Batman Turkey

**Keywords:** Acetone, aldol reaction, computational modelling, enantioselectivity, organocatalysis, proline, substituted aldehydes

## Abstract

This work involves a facile synthesis of three (S) -proline-based organocatalysts with C2 symmetry and their effects in enantioselective aldol reaction of acetone with substituted aromatic aldehydes. Moderate enantioselectivities (up to 61% ee) were obtained depending on the nature of the substituents on the aryl ring. Computational calculations at HF/6-31 + G(d) level were employed to underline the enantioselectivity imposed by all the organocatalysts. Higher calculations at B3LYP/6-311 ++ G(d,p) scrf=(solvent=dichloromethane)//B3LYP/6-31 + G(d) levels of theory were also performed for the aldol reaction of acetone with benzaldehyde and 4-nitrobenzaldehyde catalyzed by 1. The computational outcomes were consistent with those produced by experimental results and they were valuable to elucidate the mechanism for the observed stereoselectivity.

## 1. Introduction

The valuable role of nature is recognized in the selectivity and activity in biochemical processes occurring in cells. This phenomenon has inspired scientists to develop small synthetic models that could rival those presented by nature. Remarkable success has been achieved in the fields of both molecular recognition [1] and catalysis [2–4]. Enantioselective organocatalysis developed as an alternative to those involving metal ions and has become the focus of current research [5–8] after a long history of proline-catalyzed intramolecular aldol reaction of an achiral triketone with high enantioselectivity, a pioneering reaction developed in the 1970s, called the Hajos–Parrish–Eder–Sauer–Wiechert reaction [9,10].

The C-C bond-forming reactions are very important in synthetic organic chemistry since they are involved in constructing the frame of very complex molecules. The aldol reaction [11] is one of the most outstanding methods among the C-C bond-forming reactions, especially for building the hydroxyl ketones mostly found in many biologically active compounds and drugs. Thus, the design of efficient organocatalysts for asymmetric aldol reactions constitutes a significant and active area in the field of synthetic organic chemistry in recent years [12–22]. Since the discovery of the Hajos–Parrish–Eder–Sauer–Wiechert reaction, proline has occupied a key point in the development of organocatalysts and therefore many chiral organocatalysts have been discovered for aldol reactions [23–31]. The main requirements in designing these types of catalysts are the proline frame and a hydrogen donor. The current study involves the employment of three organocatalysts (1–3) in the asymmetric aldol reactions of acetones with substituted benzaldehydes. The synthesis of organocatalysts has already been reported [32–36] and they have been used in a similar manner [32,33,35,37]. These systems provide some advantages. The first is that they can easily be prepared from (S) -proline. The second is that they are highly stable because of the robust amide linkage, which can be recovered and reused without detrimental effects. Finally, they have acidic NH protons, which can act as electrophiles via hydrogen bonding to stabilizethe transition states involving negative charges.

Quantum mechanical (QM) calculations have been widely employed in the elucidation of mechanisms of asymmetric organocatalysis [38]. We have also performed QM calculations at the HF/6-31 + G(d) level to rationalize the enantioselective aldol reactions between acetone and benzaldehyde catalyzed by organocatalysts 1–3. Higher level calculations at B3LYP/6-311 ++ G(d,p) scrf=(solvent=dichloromethane)//B3LYP/6-31 + G(d) were performed for the reactions catalyzed by 1. These calculations may provide some insights to choose different reactions to achieve much better enantiomeric selectivity without experimental studies. For example, calculations at the HF/6-31 + G(d) level indicated that much better enantiomeric selectivity may be gained for the Mannich reaction of ethyl glyoxylate N -phenyl imine and formaldehyde catalyzed by 1.

## 2. Materials and methods

### 2.1. Experimental section

#### 2.1.1. General

Chemicals, unless their synthesis is mentioned, and solvents were obtained from commercial sources. Catalyst 3 was received as a gift from Dr. Mehmet Karakaplan [36]. Melting points were determined with a GALLENKAMP apparatus with open capillaries and are uncorrected. Infrared spectra were recorded on a MIDAC-FTIR Model 1700 spectrometer. The elemental analyses were obtained with a CARLO-ERBA Model 1108 apparatus. Optical rotations were taken on a PerkinElmer 341 Model polarimeter. ^1^H (400 MHz) NMR, ^13^C (100 MHz) NMR, and two-dimensional NMR (DEPT, COSY, HETCOR, HMQC, HMBC) spectra were recorded on a BRUKER DPX-400 High Performance Digital FT-NMR spectrometer in the solvents indicated. Chemical shifts are expressed in parts per million (δ) using residual solvent protons as the internal standards.

**General procedure for catalytic aldol reactions**

To a stirred solution of a catalyst (10%–20%) in a specific solvent was added aldehyde (0.25 mmol) and acetone (1.25 mmol) in the presence of an additive (10% mol) at –10 to 25 °C. The solution was kept stirring for 24–72 h (Table 1). The reaction mixture was quenched with saturated ammonium chloride solution and extracted with ethyl acetate (3 ×10 mL). The organic layer was washed with water and dried on MgSO_4_. After evaporation of the solvent under reduced pressure, the crude product was purified by column chromatography over silica gel using 1:3 ethyl acetate and hexane as an eluent to obtain the aldol products. All the aldol products are known compounds and they did not need further structural analyses. The ee values for each reaction were determined by HPLC analysis on a chiral HLPC column. The HPLC conditions and retention times are provided in Table 2.

**Table 1:** Determination of optimum conditions for the reaction of 4-nitrobenzaldehyde with acetone catalyzed by 1–3 for a period of 24–72 h.

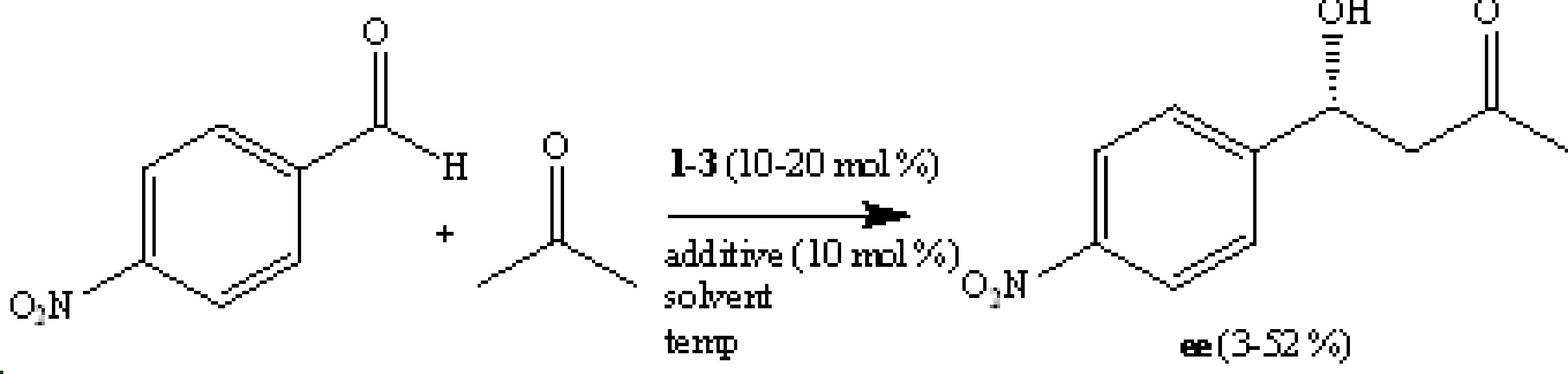							
			Retention time (min)			
1–3	R	Products	R	S	Yield (%)b	ee (%)c
**1**	10	H_2_O	-	25	24	75	11
**1**	10	DMSO	-	25	24	85	31
**1**	10	DCM	-	25	24	90	46
**1**	10	Acetone	-	25	24	90	10
**1**	10	DCM	-	2	36	90	50
**1**	10	DCM	DNP	2	36	90	40
**1**	10	DCM	PALA	2	36	90	27
**1**	10	DCM	BZA	2	36	90	52
**1**	10	DCM	BZA	-10	36	90	15
**1**	20	DCM	BZA	2	36	90	52
**2**	10	DCM	DNP	2	36	90	15
**2**	10	DCM	BZA	2	36	90	13
**2**	10	DCM	PALA	2	36	90	3
**2**	10	DCM	DNP	-10	48	90	9
**2**	10	Acetone	DNP	-10	48	90	30
**2**	10	MeOH	DNP	-10	72	90	25
**3**	10	DCM	-	2	36	90	40
**3**	10	DCM	DNP	2	36	90	17
**3**	10	DCM	PALA	2	36	90	50
**3**	10	DCM	BZA	2	36	90	52
**3**	10	DCM	BZA	-10	48	90	40
**3**	20	DCM	BZA	2	36	90	52

Reaction conditions: 4-Nitrobenzaldehyde (0.25 mmol), acetone (1.25 mmol). Reaction yield was determined based on aldol products and ee (%) was determined by HPLC (column AS-3). DCM = Dichloromethane; DNP = 2,4-dinitrophenol; BZA = benzoic acid; PALA = palmitic acid.

**Table 2:** Enantioselective aldol reactions of acetone with substituted benzaldehydes catalyzed by organocatalysts (1–3).

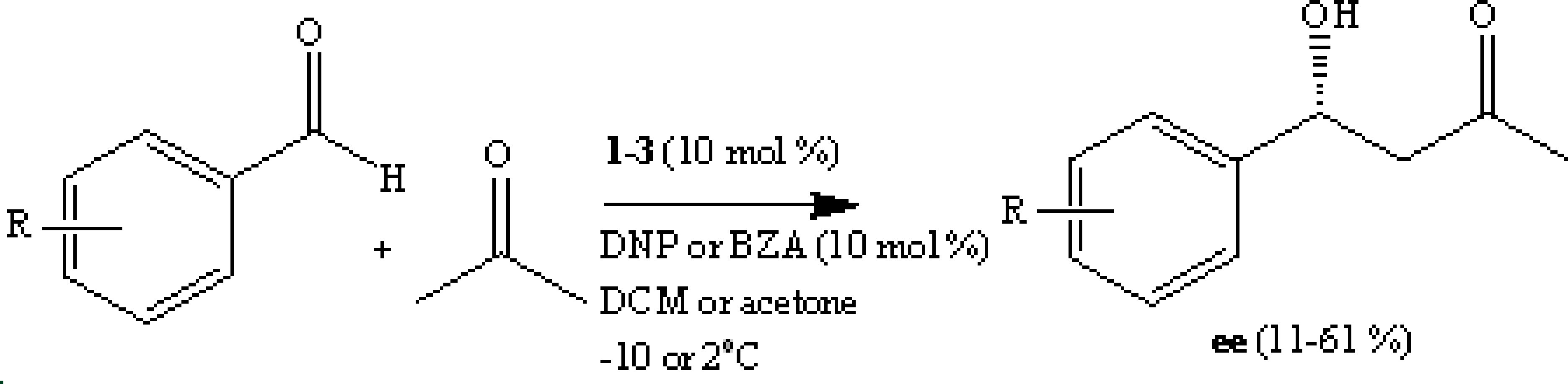							
			Retention time (min)			
1–3	R	Products	R	S	Yield (%)b	ee (%)c
**1**	4-NO_2_	4-Hydroxy-4-(4-nitrophenyl)butan-2-one	24.3	31.3	91	52
**1**	4-Cl	4-(4-Chlorophenyl-4-hydroxybutan-2-one	12.1	15.5	71	17
**1**	2-NO_2_	4-Hydroxy-4-(2-nitrophenyl)butan-2-one	14.1	9.8	85	37
**1**	2-Cl	4-(4-Chlorophenyl-4-hydroxybutan-2-one	16.5	11.8	65	11
**2**	4-NO_2_	4-Hydroxy-4-(4-nitrophenyl)butan-2-one	24.3	31.3	91	30
**2**	4-Cl	4-(4-Chlorophenyl-4-hydroxybutan-2-one	12.1	15.5	71	17
**2**	2-NO_2_	4-Hydroxy-4-(2-nitrophenyl)butan-2-one	14.1	9.8	85	45
**2**	2-Cl	4-(4-Chlorophenyl-4-hydroxybutan-2-one	16.5	11.8	65	19
**3**	4-NO_2_	4-Hydroxy-4-(4-nitrophenyl)butan-2-one	24.3	31.3	95	52
**3**	4-Cl	4-(4-Chlorophenyl-4-hydroxybutan-2-one	12.1	15.5	70	19
**3**	2-NO_2_	4-Hydroxy-4-(2-nitrophenyl)butan-2-one	14.1	9.8	85	61
**3**	2-Cl	4-(4-Chlorophenyl-4-hydroxybutan-2-one	16.5	11.8	65	40

Reaction conditions catalyzed by 1: Catalyst (0.025 mmol), substrates (0.25 mmol), acetone (1.25 mmol), solvent = DCM (0.5 mL) and additive = BZA (0.025 mmol) at 2 °C; by 2: catalyst (0.025 mmol), substrates (0.25 mmol), acetone (1.25 mmol), solvent = acetone (0.5 mL) and additive = DNP (0.025 mmol) at –10 °C; by 3: catalyst (0.025 mmol), substrates (0.25 mmol), acetone (1.25 mmol), solvent = DCM (0.5 mL) and additive = BZA (0.025 mmol) at 2 °C.

#### 2.1.2. Synthesis

**(2S,2S)-Di-tert-butyl-2,2-{[pyridine-2,6-diylbis(azanediyl)]bis-(carbonyl)}bis(pyrrolidine-1 carboxylate) (1a)**

A mixture of 2,6-diaminopyridine (1.15 mmol, 125 mg) and Boc-L-proline (2.3 mmol, 495 mg) in 10 mL of DCM was placed in a round-bottomed flask (50 mL) in an ice bath. Dicyclohexylcarbodiimide (DCC) (2.3 mmol, 473 mg) was added to the mixture via a funnel within 30 min. The mixture was kept stirring overnight. The participated dicyclohexylurea was removed by filtration and DCM (50 mL) was added to the remaining solution. The solution was washed with 5% citric acid (3 ×15), NaHCO3 (3 ×15 mL), and water and dried on MgSO_4_. After evaporating the solvent the remaining residue was purified by column chromatography on silica using ethyl acetate and methanol (50:1), affording 170 mg of 1a (60%). Mp 79–81 °C (literature [32]: 77–80 °C), [α]^21^_D_: –79.0 (c = 1, CH_2_Cl_2_) (literature [32]: [α]^23^_D_: –107.5 (c = 0.23, CH_2_Cl_2_). Chemical analysis calculated for C_25_ H_37_ N_5_ O_6_ : C 59.63; H 7.41; N 13.91; found: C 59.60; H 7.40; N 13.90. ^1^H NMR (400 MHz, δ ppm, CDCl_3_) : 9.27 (br, 2H, NH), 8.21 (br, 2H, NH), 7.91 (d, 2H, H-Py, J = 7.6 Hz), 7.69 (br, 1H, H-Py), 4.47–4.29 (m, 2H, CHN), 3.58 (br, 4H, NCH_2_), 2.43–1.94 (m, 8H, CH_2_) , 1.43 (s, 18, CH_3_). ^13^C NMR (100 MHz, δ ppm, CDCl_3_): 171.26, 170.53, 149.29, 140.60, 109.48, 80.96, 62.11, 47.19, 33.96, 30.98, 28.33. IR (KBr disc cm^-1^): 3343, 2972, 2930, 1688, 1545, 1441, 1154, 1116, 930, 804.

**(2S,2S)-2,2-[Pyridine-2,6-diylbis(azanediyl)]bis(pyrrolidine-2-carboxamide)(1) 1a**

(0.55 mmol, 0.28 g) in DCM (5 mL) in an ice-bath was completely dissolved and then TFA and AcOH (1:1) (631 μL) were added. This solution was stirred overnight at room temperature. The organic solvent was evaporated under reduced pressure and 1 N NaOH (25 mL) was added to the remaining aqueous solution, following by extraction with DCM (3 ×15 mL), and dried on Na2 SO4 . The solvent was removed by evaporation under reduced pressure, affording 178 mg of a yellowish solid (98%). Mp 165–167 °C (literature [33]: mp 115–117 and 177.5–177.9 °C). [α]18.5 D : +10.8 (c = 0.25, CH_2_Cl_2_) . Literature [32,33]: ([α]^23^_D_= –117.5 (c = 0.11 in CH_2_Cl_2_) [32] and ([α]^23^_D_= +10.0 (c = 0.1 in CHCl3) [33]. Chemical analysis calculated for C15 H21 N_5_ O2 : C 59.39; H 6.98; N 23.09; found: C 59.36; H 6.906; N 23.07. ^1^H NMR (400 MHz, δ ppm, CDCl_3_) : 9.99 (s, 2H, NH), 7.95 (d, 2H, H-Py, J = 7.8), 7.70 (t, 1H, H-Py, J = 7.8 Hz), 3.89–3.86 (m, 2H, CHN), 3.10–3.05 (m, 4H, CH_2_ N), 2.23–1.77 (m, 10H, CH_2_ CH_2_ and NH). ^13^C NMR (100 MHz, δ ppm, CDCl_3_) : 174.18, 149.43, 140.56, 109.09, 61.11, 47.36, 30.86, 26.28. IR (KBr disc cm^-1^) : 3319, 3208, 2928, 2851, 1688, 1583, 1491, 1446, 1293, 1110,795.

**(2S,2S)-Di-tert-butyl 2,2-{[1,3-phenylenebis(methylene)]bis(carbonyl)}bis(pyrrolidine-1 carboxylate) (2a)**

Boc-L-proline (4.6 mmol, 0.99 g) was dissolved in dry dichloromethane (DCM) (10 mL) and the solution was cooled down to 0 °C in an ice-salt bath, followed by adding 1-(3-dimethylaminopropyl)-3-ethylcarbodiimide hydrochloride (EDCl) (4.6 mmol, 0.90 g) and 1-hydroxybenzotriazole monohydrate (HOBt) (4.6 mmol, 0.7 g), respectively. Then 1,3-benzenedimethanamine (2.3 mmol, 312 mg) in dry DCM (5 mL) was added to the mixture via a syringe within 15 min at 0 °C. The mixture was kept stirring at rt for 24 h. The mixture was filtered and the solvent was evaporated under reduced pressure. The remaining residue was dissolved in chloroform (50 mL) and washed with water (2 ×20 mL) and saturated sodium bicarbonate (3 ×15 mL), respectively, and dried on Na2 SO4 . After evaporating the solvent, the residue was crystallized in ethyl acetate (250 mg, 65%). Mp 171–172 °C, [α]^21^_D_: –65.2 (c 1, CH_2_Cl_2_) . Chemical analysis calculated for C28 H42 N4 O_6_ : C 63.37; H 7.98; N 10.56; found: C 63.35; H 7.95; N 10.54. ^1^H NMR (400 MHz, δ ppm, CDCl_3_) : 7.28–7.17 (m, 4H, Ar-H), 6.42 (br, 2H, NH), 4.36 (br, 6H, CH_2_ Ar and CHN), 3.45 (br, 4H, NCH_2_) , 1.96–1.90 (m, 8H, CH_2_) , 1.44 (s, 18H, CH_3_) . ^13^C NMR (100, MHz, δ ppm, CDCl_3_) : 172.44, 138.74, 129.05, 126.70, 80.46, 60.07, 47.14, 43.14, 28.32, 24.97, 24.65. IR (KBr disc cm^-1^) : 3313, 2976, 2927, 1682, 1552, 1398, 1123, 891.

**(2S,2S)-2,2-[1,3-phenylenebis(methylene)]bis(pyrrolidinecarboxamide) (2) 2a**

(0.55 mmol, 0.3 g) in DCM (5 mL) in an ice bath was completely dissolved and then TFA and AcOH (1:1; 631 μL) were added. This solution was mixed overnight at room temperature. The organic solvent was evaporated under reduced pressure and 1 N NaOH (25 mL) was added to the remaining aqueous solution, following by extraction with DCM (3 ×15 mL) and drying on Na2 SO4 . The solvent was removed by evaporation under reduced pressure, affording 170 mg (93%) of a yellowish viscose material. Chemical analysis calculated for C18 H26 N4 O2 : C 65.43; H 7.93; N 16.96; found: C 65.45; H 7.95; N 16.94. ^1^H NMR (400 MHz, δ ppm, CDCl_3_) : 8.11 (s, 2H, NH), 7.28–7.13 (m, 4H, Ar-H), 4.39 (d, 4H, CH_2_ -Ar, J = 6.0 Hz), 3.85–3.82 (m, 2H, CHN), 3.03–2.97 (m, 4H, NCH_2_) , 2.93–2.87 (m, 2H, NH), 2.21–2.11 and 1.97–1.90 (2m, 4H, CH_2_) , 1.89–1.64 (m, 4H, CH_2_ CH_2_) . ^13^C NMR (100 MHz, δ ppm, CDCl_3_) : 175.01, 139.05, 128.90, 126.63, 126.45, 60.64, 47.19, 42.93 (Ar-CH_2_) , 30.92 (CH_2_ CH), 26.12 (CH_2_ -CH_2_) . IR (KBr disc cm^-1^) : 3287, 3061, 2927, 2868, 1645, 1514, 1243, 1110, 699.

### 2.2. Computational modeling

Molecular dynamic (MD) calculations were carried out to obtain the conformational changes in enamines. MD simulations were run using the AMBER (version 9.0) suite of programs [39]. The compounds were designed with GaussView 3.09 [40] and optimized with Gaussian 03 [41] using the semiempirical AM1 method [42]. AM1-Bcc (Austian model with Bond and charge correction) [43,44] atomic partial charges for the catalysts were determined by the antechamber module of the AMBER package. The general AMBER force field (GAFF) [45] was used for the MD simulations. Each molecule was minimized with a total of 5000 steps, 2500 of steepest descent followed by 2500 of conjugate gradient (maxcyc – ncyc), using a nonbonded cutoff of 999 Å in vacuum (igb? =? 0). The system was then heated from 0 to 700 K in 14 steps for a period of 350 ps and further simulated at 700 K for a period of 50,000 ps (igb? = ?0). Cluster analysis was performed with 100 intervals out of 50,000 frames to obtain a conformer with a larger population to represent the lower energy conformer. Chimera (UCSF) [46] was employed to display 3D structures and to obtain cluster analyses. The conformer with the lowest energy was used as a template, where the bond between enamine and aldehyde was scanned by HF/6-31 + G(d) to obtain the transition state without any restraints. The maximum point for each scan was used for the transition state (TS) search. TS structures were also calculated at B3LYP/6-31 + G(d) for the aldol reactions of acetone with substituted benzaldehydes catalyzed by catalyst 1 bearing only one enamine residue at the catalytic site. The single-point energy calculations were carried out for the TS structures derived from the reactions of acetone with benzaldehyde and 4-nitrobenzaldehyde catalyzed by 1 at B3LYP/6-311 ++ G(d,p) scrf = (solvent=dichloromethane)//B3LYP/6-31 + G(d).

## 3. Results and discussion

### 3.1. Synthesis

The structures of the compounds were elucidated by IR, ^1^H, and ^13^C NMR spectra. In addition, C, H, and N analyses were performed for all compounds. Organocatalysts 1 (see Figure S1 for its ^1^H and ^13^C NMR spectra) and 2 were obtained in high yields and purity by the acid-catalyzed hydrolysis of their precursors (1a and 2a). These precursors were prepared from the straightforward reaction of (S) -N -Boc-proline with diamino linkers (2,6-diaminopyridine and 1,3-benzenedimethanamine) in good yields (Scheme). Precursor 1a was synthesized in 60% yield by reaction of an excess of (S) -Boc-proline with 2,6-diaminopyridine. The synthesis of this precursor and the corresponding deprotected amine has recently been reported using different coupling agents. However, different melting points and specific optical rotations were reported for the same compounds [32,33]. Here we employed dicyclohexylcarbodiimide (DCC) as a coupling agent and the compound was obtained without applying column chromatography. The synthesis of organocatalyst 2 was reported as a patent [34]. Here we used 1-(3-dimethylaminopropyl)-3-ethylcarbodiimide hydrochloride (EDCl) and 1-hydroxybenzotriazole monohydrate (HOBt) as coupling reagents to prepare precursor 2a with a yield of 65%. The deprotection of 2a by a 1:1 mixture of DCM/TFA at 25 °C overnight gave corresponding proline-based organocatalyst 2 (Scheme). Compound 3 [35] was obtained by a previous group [36].

**Scheme:**
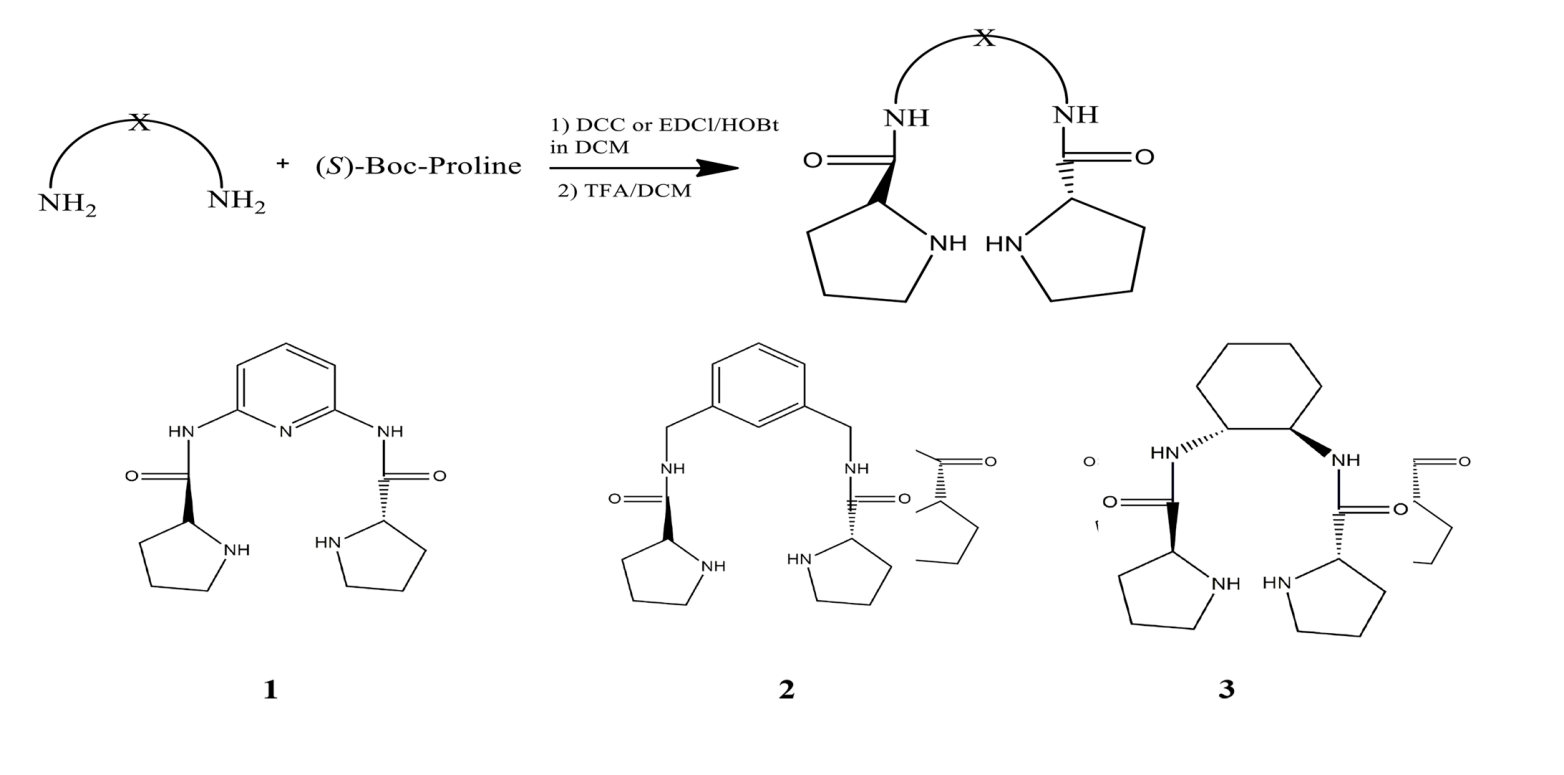
Catalysts (1-3) prepared from the straightforward reaction of (S)-N -Boc-proline with diamino linkers.

### 3.2. Catalysis

In order to determine the enantiomeric excesses of the organocatalysts, the reaction of each substituted benzaldehyde with acetone was first carried out according to the literature methods in the absence of organocatalysts [47]. The optimum column and solvent system was determined for separation of the enantiomers from these racemic mixtures. Conditions for the separation of racemic mixtures are presented in Table 2. These data show that racemic mixtures of all reactions are successfully separated under these conditions (see Figure S2 and Figure S3 for GC chromatograms of enantiomers derived from the aldol reaction of acetone with 4-nitrobenzaldehyde in the absence and in the presence of organocatalyst 1).

The catalytic effects of organocatalysts on aldol reactions of acetone with substituted benzaldehydes were investigated by changing various parameters to determine the optimum conditions for the catalyzed reactions. These parameters are the amount of organocatalysts, reaction time, temperature, solvent, and additive substance, respectively. The data are summarized in Table 1. It was observed that organocatalysts 1 (10% mol) and 3 (10% mol) catalyzed the corresponding reaction with the highest ee (52%) in DCM with benzoic acid as additive at 2 °C while catalyst 2 (10% mol) gave the highest ee (30%) in acetone with DNP as an additive at –10 °C. The reactions of acetone with other substituted aldehydes were thus carried out under these optimum conditions for each organocatalyst (1–3) as demonstrated in Table 2.

**Figure 1:**
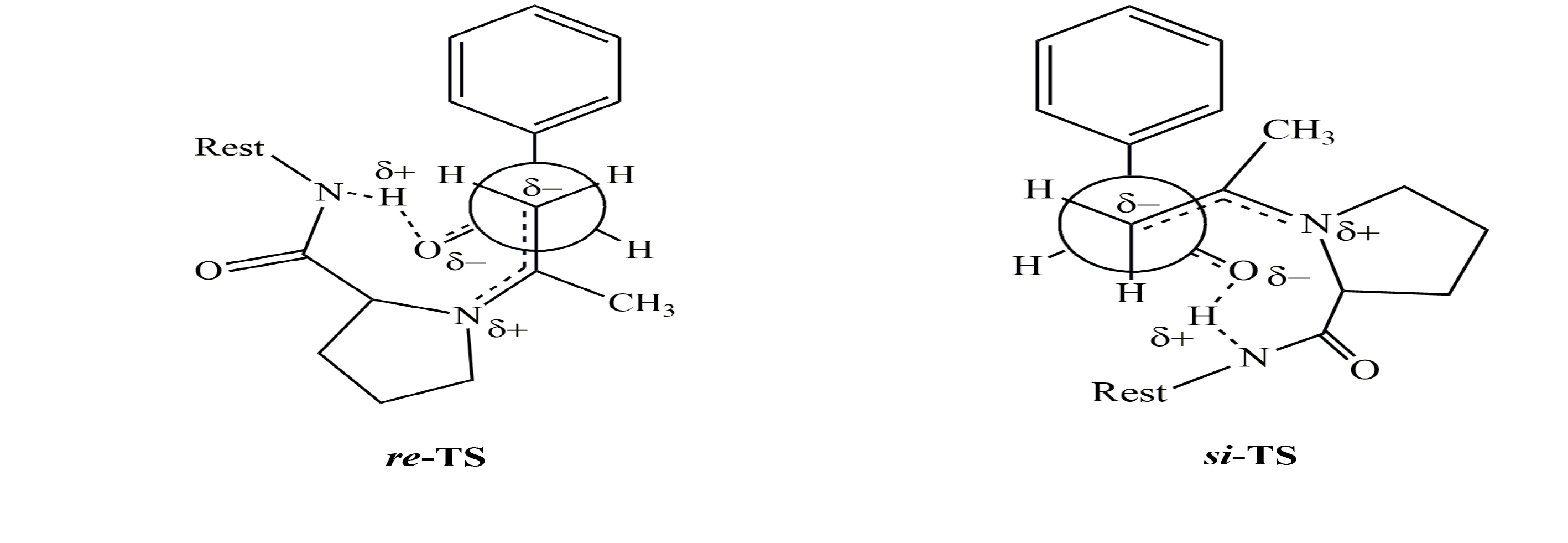
A proposed transition state structure of aldol reaction of acetone with benzaldehyde. (left) The attack of methane group on re-face leading to the formation of R enantiomer. (right) The attack of methane group on si-face leading to the formation of S enantiomer.

Experimental data show that the aldol reactions of acetone with substituted benzaldehydes catalyzed by organocatalysts 1–3 favor the formation of 4-hydroxy-4-substituted phenylbutan-2-one with R configuration. The R enantiomer is formed via the attack of the methine group on the re-face of the carbonyl group of aldehydes while the attack on the si-face would lead to the formation of the S enantiomer. A model (Figure 1) is proposed to rationalize the enantiomeric discrimination of aldol reactions of acetone with aldehydes catalyzed by the organocatalysts (1–3). The reason for better discrimination of 1 and 3 compared to 2 may be the more flexible structure of 2 due to the methane linkage between the amide function and the phenyl ring.

It is well established that the ring size and the presence of a carboxyl group in proline play an essential role in asymmetric aldol reactions [48]. Aldol reaction of acetone with p-nitrobenzaldehyde catalyzed by L−proline proceeds with 68% yield and 76% ee [48]. Here we assumed that NH-amide functions would act in a similar manner by providing electrophilic assistance to the negatively charged carbonyl group of aldehydes developing in the transition states through hydrogen bonding. The enantioselectivity obtained by the reaction of acetone with aldehydes catalyzed by 1–3 is lower compared with that catalyzed by L−proline. However, the selectivity is quite comparable for the aldol reaction of cyclohexanone with aldehydes by the same catalyst (1) [32]. It is believed that the hydrogen bonding involving the proline acid group and the aldehyde is important for the high enantioselectivity [49,50].

### 3.3. Computational modeling

**Figure 2:**
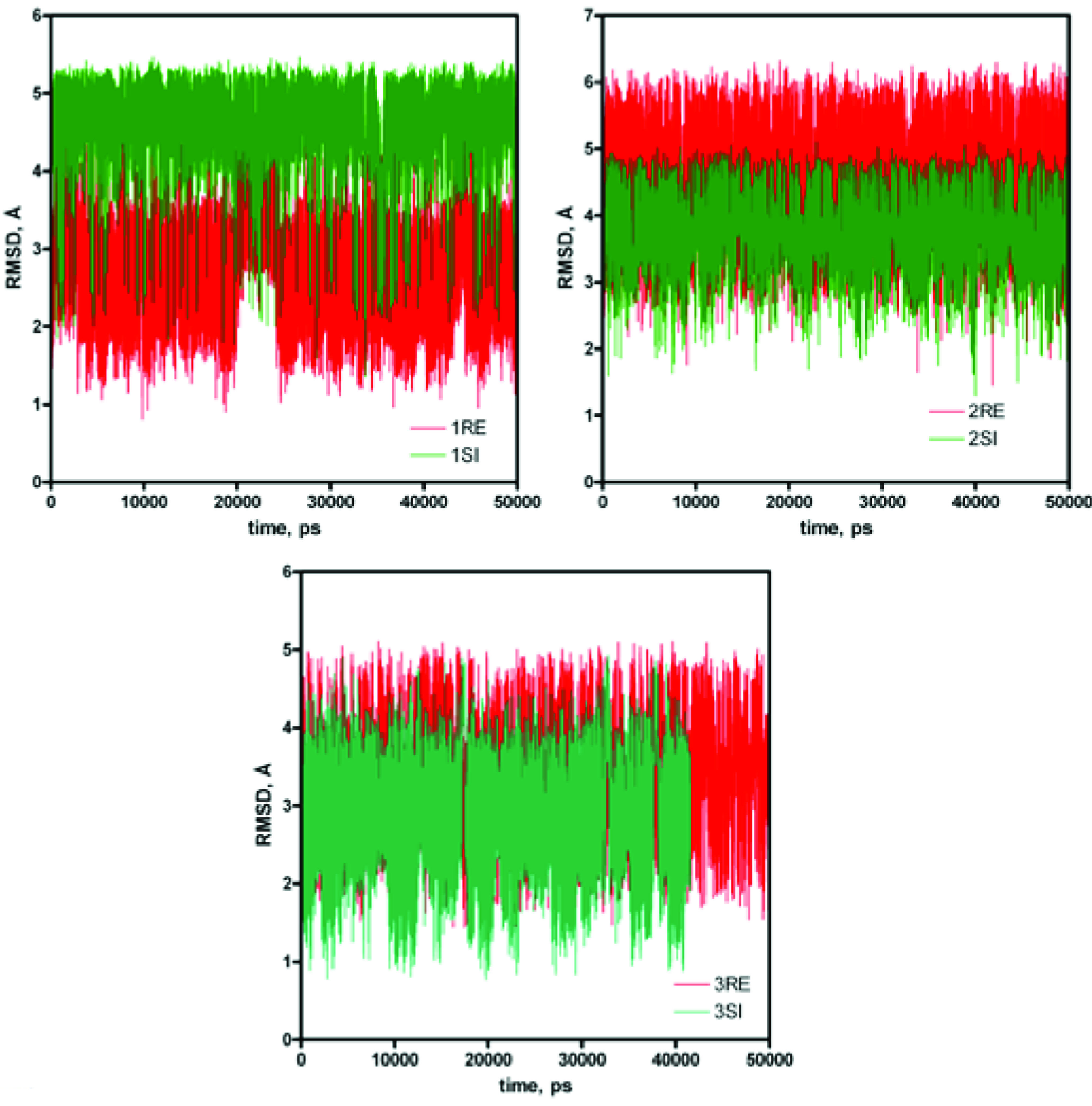
RMSD changes for the intermediates derived from the reaction of enamine derivatives of catalysts (1–3) with benzaldehyde during MD calculation for a period of 50 ns in vacuum.

**Figure 3:**
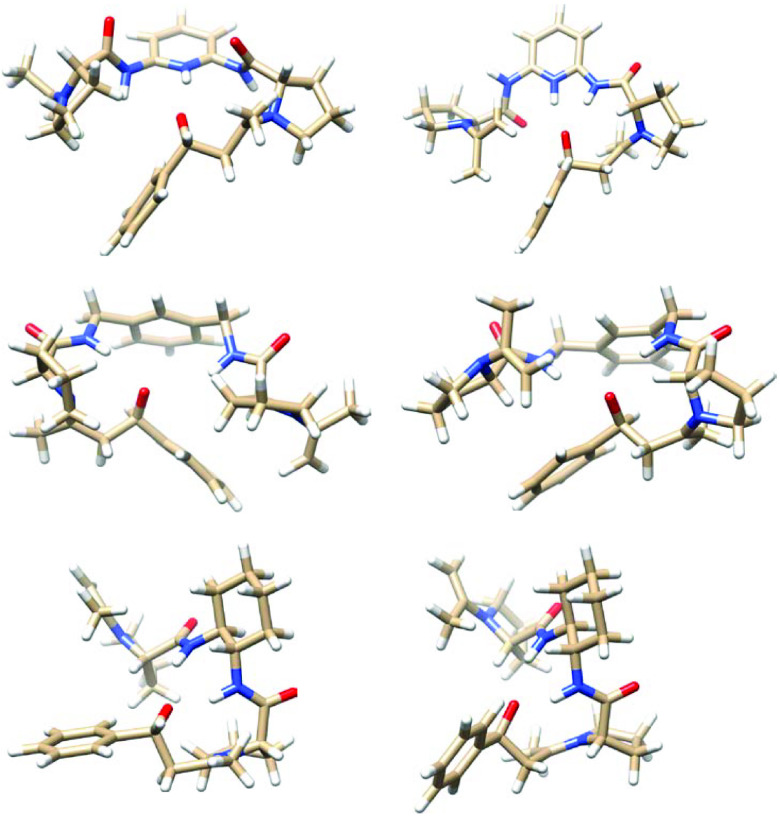
The lower energy conformers (right for the si-face and left for the re-face) for the tetrahedral intermediates of the reaction of enamine derivatives of catalysts (1–3) with benzaldehyde. From the top to the bottom for the reaction of 1, 2, and 3, respectively.

The source of the enantiomeric excess was successfully modeled in a previous study [37]. For this kind of calculation, it is important to start with an accurate structure to determine the structures of transition states. The source of enantiomeric excess will be reflected in the difference in the energies of transition states for the reaction of enamine formed between the organocatalysts and acetone with benzaldehydes. These reactions would proceed through a tetrahedral intermediate, so we assumed that the transition states would resemble these intermediates. Therefore, the conformational analysis of these compounds will produce a reasonable standpoint to predict the transition states for these reactions. Molecular dynamic calculations provide insightful information about conformation changes in a molecule. The conformation changes in the tetrahedral intermediates derived from the reaction of enamines with benzaldehydes were analyzed by MD calculations. The root mean square deviation (RMSD) for each intermediate during MD calculations is shown in Figure 2. It shows that all the structures largely prefer one conformer with a highest population. The conformer for each intermediate is obtained from the cluster analysis of MD trajectories (Figure 3). The lower energy conformers derived from the cluster analyses offer a good standpoint for transition state searches. They all involve the hydrogen bond between the amide functions in the catalysts and the negatively charged hydroxyl group in the aldehydes. The intermediate in the case of the reaction catalyzed by catalyst 1 also involves a hydrogen bond between the pyridinium function and hydroxyl group (Figure 3).

**Figure 4:**
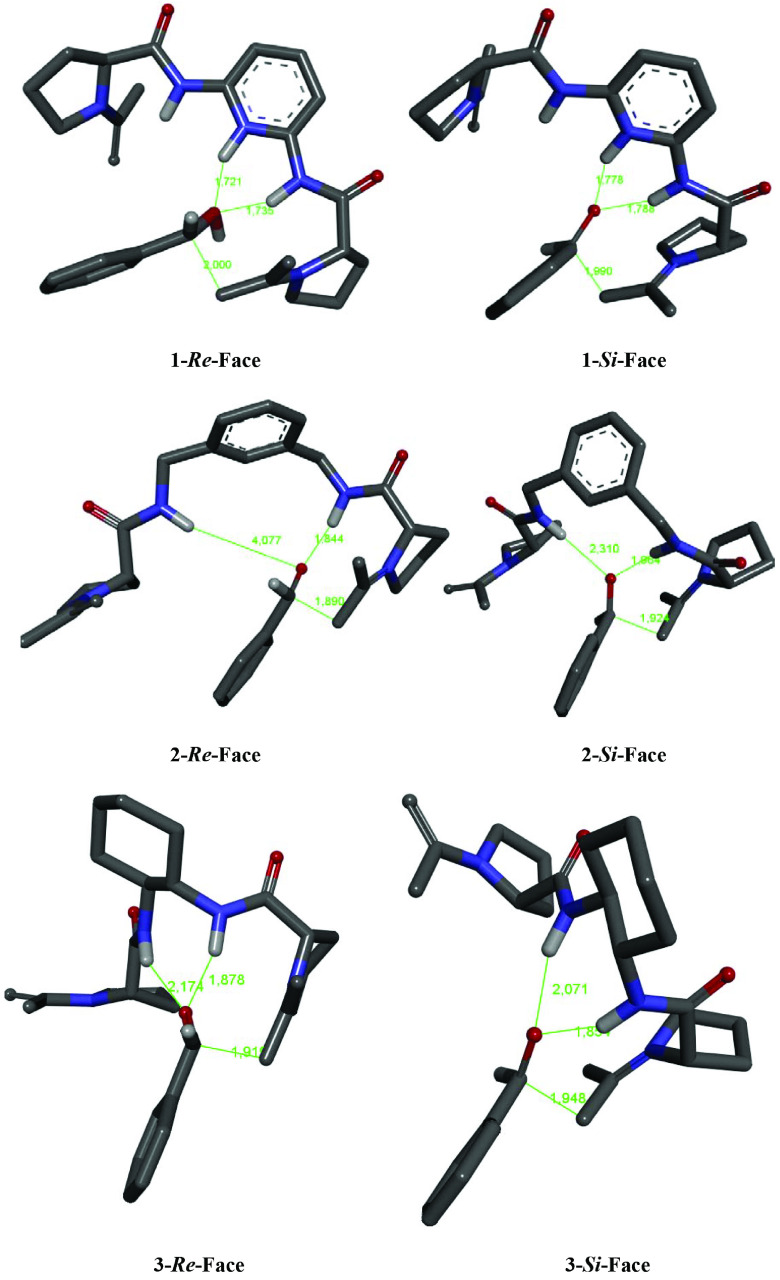
The transition states calculated at HF/6-31 + G(d) for the aldol reaction of acetone with benzaldehyde leading to the formation of the enantiomers of 4-hydroxy-4-phenylbutan-2-one catalyzed by organocatalysts 1–3. Discovery Studio Visualizer 4.1 was used to display the structures [51].

The bond between enamine and benzaldehydes in each conformer was scanned and consequently the structures with the highest energy in the reaction coordinate were selected for the transition state search. All the scanning produced a smooth curve with a summit, which was further searched for the transition state. The transition states were identified by having a negative imaginary frequency. The calculations were carried out by Hartree–Fock method at 6-31 + G(d) for the reactions catalyzed by 1–3(Table 3). The B3LYP/6-31 + G(d) level of theory was also employed for the aldol reactions of acetone with substituted benzaldehyde catalyzed by 1 with one enamine residue (Table 4). The calculations were performed at B3LYP/6-311 ++ G(d,p) scrf=(solvent=dichloromethane)//B3LYP/6-31 + G(d) levels to include the solvent effect for the reaction of acetone with benzaldehyde and 4-nitrobenzaldehyde (Table 5). All the calculations show that the reactions lead to a preferable formation of 4-hydroxy-4-(substituted phenyl)butane-2-one with R absolute configuration because the transition states leading to R enantiomers have lower energies compared to those leading to S enantiomers. Transition states leading to both enantiomers for the reaction of acetone with benzaldehyde catalyzed by 1–3 are represented in Figure 4. The bond-forming distances in the transition states of the reaction of acetone with benzaldehyde catalyzed by 1–3 leading to 4-hydroxy-4-phenylbutan-2-one are reflected in Figure 4. The results show that the transition states via the attack on the si-face leading to S enantiomers are more advanced than those via the attack on the re-face for the reaction catalyzed by 1 and 3.

**Table 3:** Calculated transition state energies at HF/6-31 + G(d) level for the aldol reaction of benzaldehyde with acetone catalyzed by **1-3** .

Catalyst	E_*re*_, Hartree	E_*si*_, Hartree	ΔE^‡^, kcal mol^-1^
**1**	–1577.93731690	–1577.93530650	1.26
**2**	–1639.58782656	–1639.58757980	0.15
**3**	–1565.01694783	–1565.01497439	1.23

**Table 4:** Calculated energies of the transition states obtained at B3LYP/6-31 + G(d) for the aldol reactions catalyzed by organocatalyst **1** with one enamine residue.

Benzaldehydes	E_*re*_, Hartree	E_*si*_, Hartree	ΔE^‡^, kcal mol^-1^
H	–1471.22215390	–1471.21937616	1.74
4-Nitro	–1675.72596387	–1675.72250216	2.17
2-Nitro	–1675.72400000	–1675.71802922	3.74
4-Chloro	–1930.81582189	1930.81243831	2.12
2-Chloro	–1930.81579944	–1930.81166636	2.59

The theoretical calculations are quite consistent with those obtained from experimental measurements in terms of enantiomeric selectivity. The better selectivity of 1 and 3 compared to 2 as observed by experimental measurements is reflected in theoretical calculations (Table 3). The energy difference calculated at HF/6-31 + G(d) for the TS of the structures leading to each enantiomer for the aldol reaction of acetone with benzaldehyde catalyzed by 1–3 is 1.26, 0.15, and 1.23 kcal mol^-1^, respectively. The energy difference obtained from the higher level of calculations at B3LYP/6-31 + G(d) for the aldol reaction of acetone with substituted benzaldehydes (Table 4) is also consistent with the experimentally observed enantiomeric excesses (Table 2). Moreover, the energy differences calculated for the TS for both HF and DFT, leading to two enantiomers for the reaction of acetone with 4-nitrobenzaldehyde, are clearly comparable with the experimental ee % values (Table 5). This obviously demonstrates the convenience of the theoretical methods applied in this study to underline the action of catalysts in these reactions.

**Table 5:** Calculated energies for the aldol reaction of 4-nitrobenzaldehyde with acetone catalyzed by **1**.

Methods	E_*re*_, Hartree	E_*si*_, Hartree	ΔE^‡^, kcal mol^-1^
HF/6-31 + G(d)	–1781.40689070	–1781.40423631	1.67
B3LYP/6-31 + G(d)	–1792.43740590	–1792.43428507	2.85
.SCRF*	–1792.93896219	–1792.93604120	1.83

*B3LYP/6-311 ++ G(d,p) scrf=(solvent=dichloromethane)//B3LYP/6-31 + G(d).

**Figure 5:**
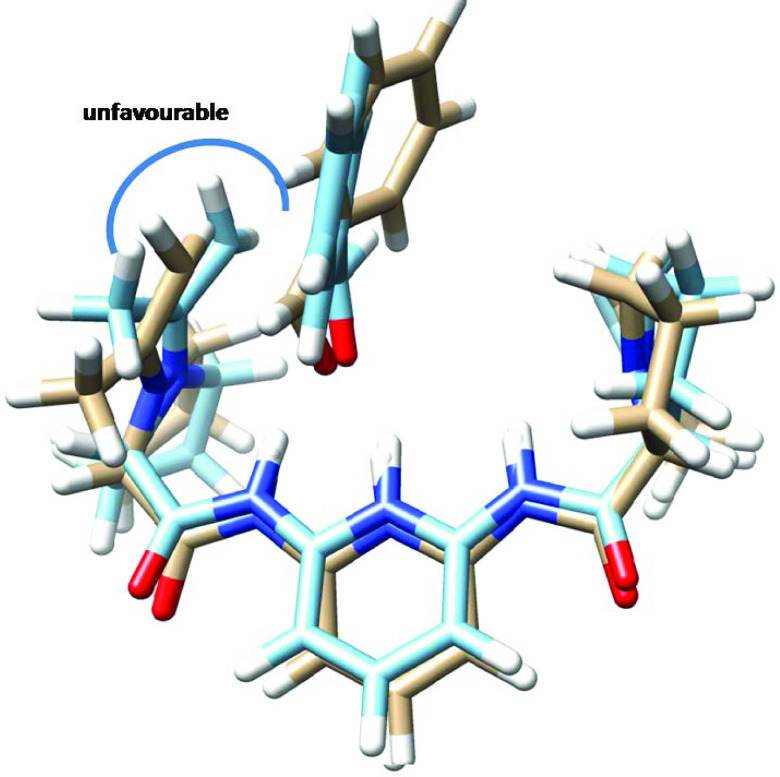
Superimposed transition states (light brown: re-face and light blue: si-face) calculated at HF/6-31 + G(d) for the aldol reaction of acetone with benzaldehyde leading to the formation of the enantiomers of 4-hydroxy-4-phenylbutan-2-one catalyzed by organocatalyst 1.

**Figure 6:**
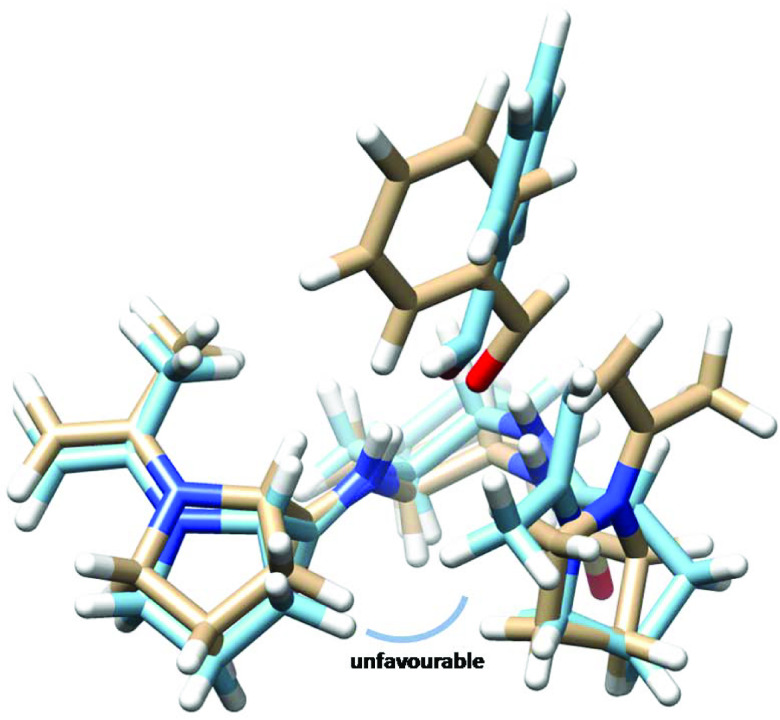
Superimposed transition states (light brown: re-face and light blue: si-face) calculated at HF/6-31 + G(d) for the aldol reaction of acetone with benzaldehyde leading to the formation of the enantiomers of 4-hydroxy-4-phenylbutan-2-one catalyzed by organocatalyst 3.

**Figure 7:**
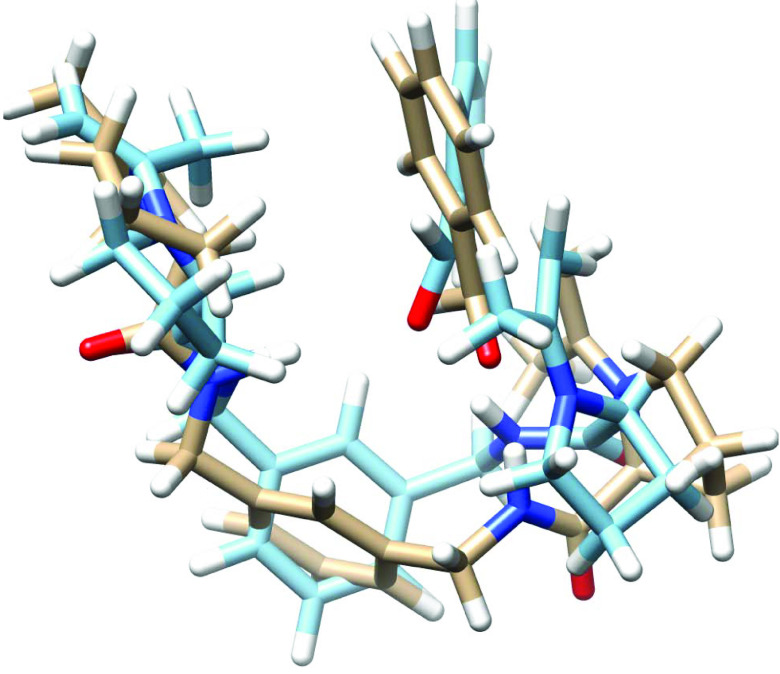
Superimposed transition states (light brown: re-face and light blue: si-face) calculated at HF/6-31 + G(d) for the aldol reaction of acetone with benzaldehyde leading to the formation of the enantiomers of 4-hydroxy-4-phenylbutan-2-one catalyzed by organocatalyst 2.

Theoretical calculations may explain the source of the experimental enantioselectivity obtained for the catalysts. The cause of enantiomeric excess exposed by 1 appears to be due to the unfavorable interaction of the methyl group of acetone in enamine with the phenyl group of benzaldehyde (Figure 5). On the other hand, the source of enantioselectivity by 3 is different than that of 1. Namely, different favorable and unfavorable interactions are involved in the catalysis by 3 (Figure 6). It is evident that there is an unfavorable interaction between the methyl group of the acetone residue in enamine with the proline ring in the other arm of the catalyst. The lower selectivity by 2 may be ascribed to the lack of specific differences in the steric or attractive interactions between the transition states leading to two enantiomers, probably because of the flexibility of the catalyst (Figure 7). This may be represented by the superimposed structures of two transition states produced from the attack of enamine at the re- and si-face of benzaldehyde (Figure 7). It demonstrates that there is no clear-cut difference between two intermediates in terms of favorable or unfavorable interactions.

**Figure 8:**
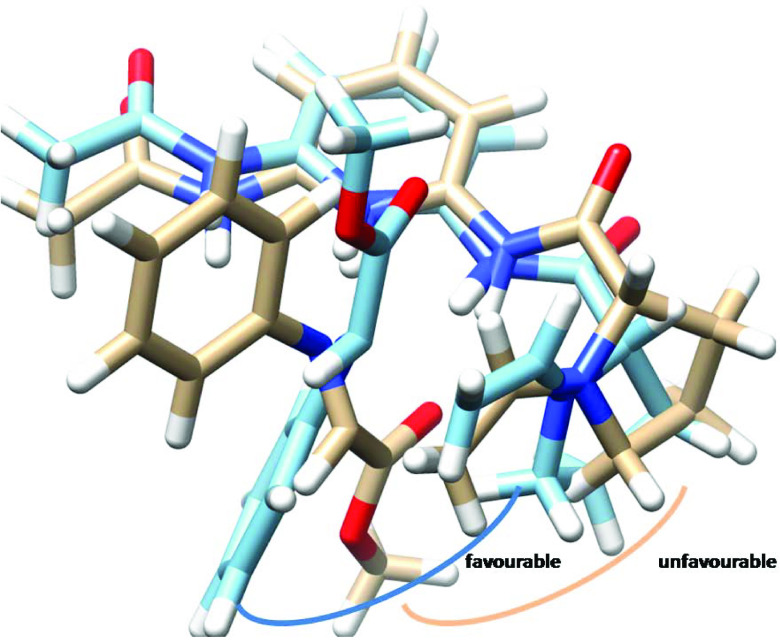
Superimposed transition states [light brown: re-face (–1458.79730136 Hatree) and light blue: si-face (– 1458.8026812^1^Hartree)] calculated at HF/6-31 + G(d) for the Mannich reaction of ethyl glyoxylate N -phenyl imine and formaldehyde catalyzed by 1.

In general, the lower selectivity of the organocatalysts (1–3) was ascribed to the lack of their specific favorable or unfavorable interactions in the transition states leading to products. Calculations at the HF/6-31 + G(d) level demonstrated that organocatalyst ^1^Has much better enantiomeric selectivity for the Mannich reaction of ethyl glyoxylate N -phenyl imine and formaldehyde catalyzed by 1 compared to that of the aldol reaction of acetone with benzaldehydes. The reaction prefers attacking at the si-face of the substrate with an energy difference of 3.78 kcal mol^-1^. This is associated with the specific interaction involving well-defined hydrogen bonds and π/CH interaction between the phenyl group in the substrate and proline ring CH in the catalyst in the preferred transition state (Figure 8). All the structures of the transition states for the reactions are given in the Supplementary information in SYBYL format.

## 4. Conclusion

Three L−proline-based organocatalysts were synthesized by the modification of available methods in the literature and their asymmetric induction effects on aldol reactions of acetone with substituted benzaldehyde were investigated. These organocatalysts were found to catalyze the corresponding reactions with up to 61% enantiomer excess. The details of reactions leading to the enantiomeric excesses were revealed by theoretical calculations. Although the enantiomeric discrimination is not large enough to compare with similar systems, the data are valuable to the field in the design of new organocatalysts. Furthermore, they may be tested in the aldol reactions of different ketones other than acetone with aldehydes.

Supplementary Materials
