# Supplementary material for: Proline-based organocatalyst-mediated asymmetric aldol reaction of acetone with substituted aromatic aldehydes: an experimental and theoretical study
Source: Turk J Chem. 2020 Apr 1;44(2):335–51. doi: 10.3906/kim-1908-3 (PMC7671204; doi:10.3906/kim-1908-3)
Supplement: Supplementary file 1 — Supplementary Materials [file turkjchem-44-335-sup001.pdf]

Supplementary information

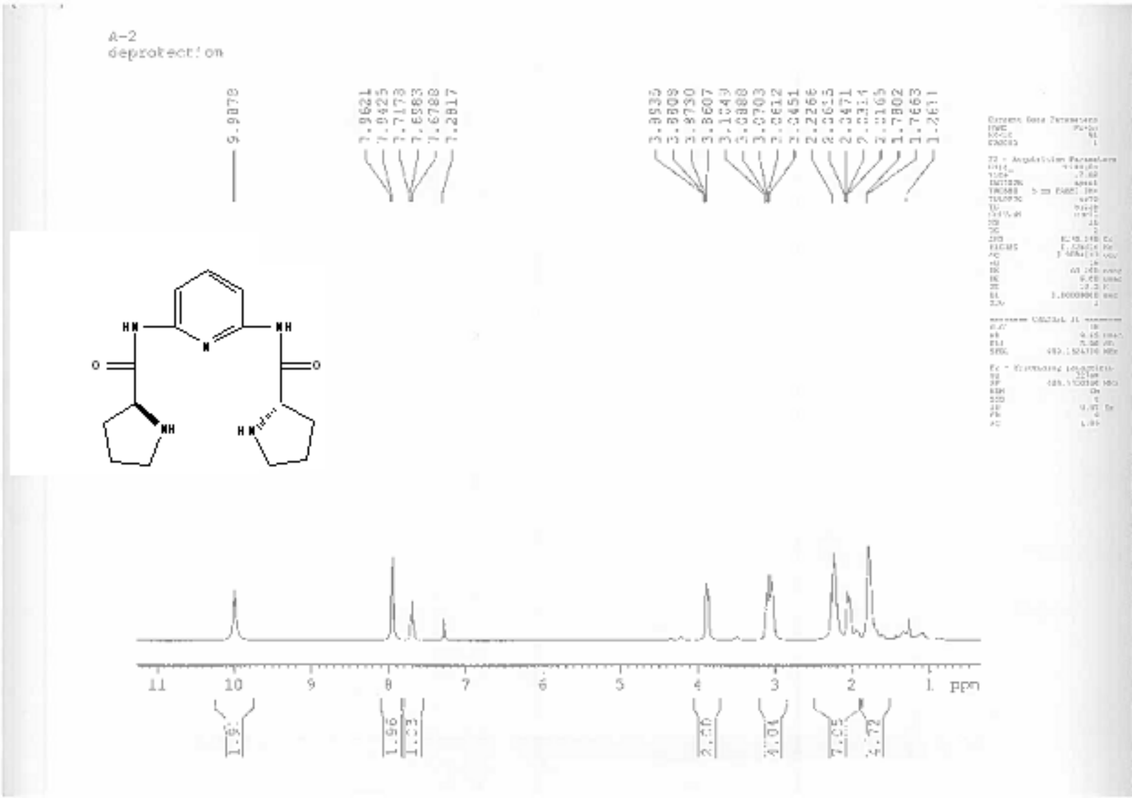

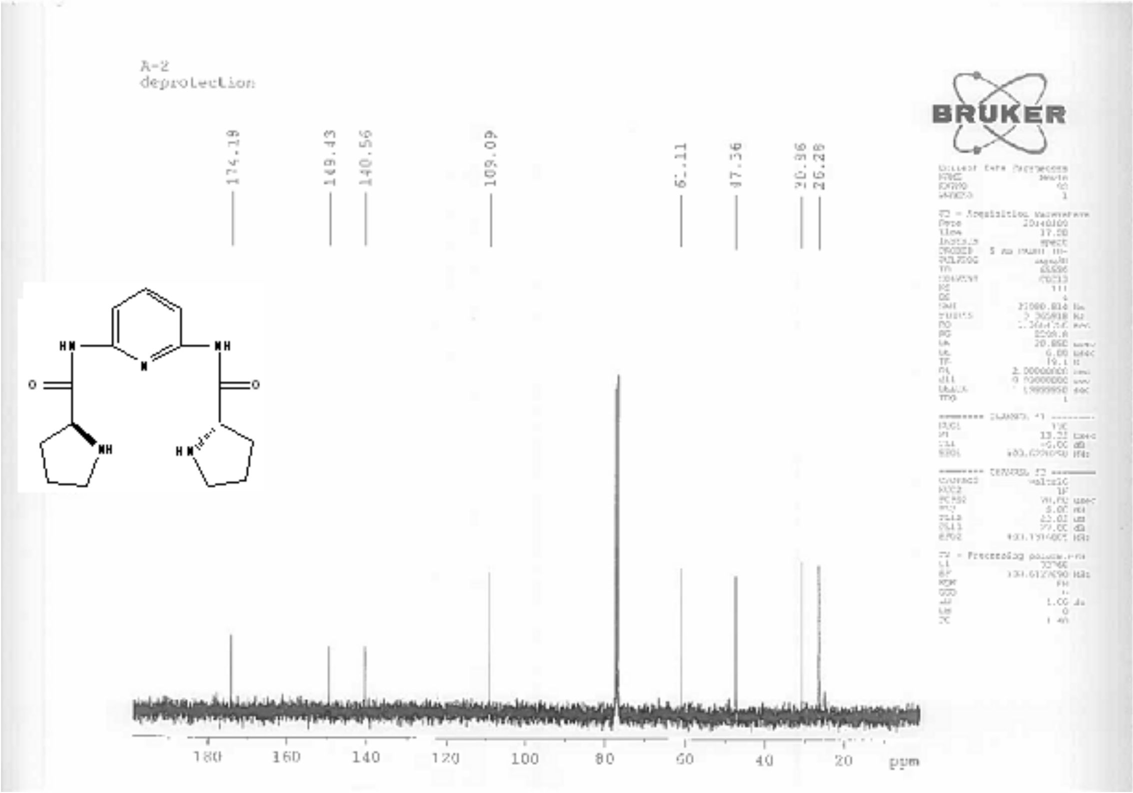

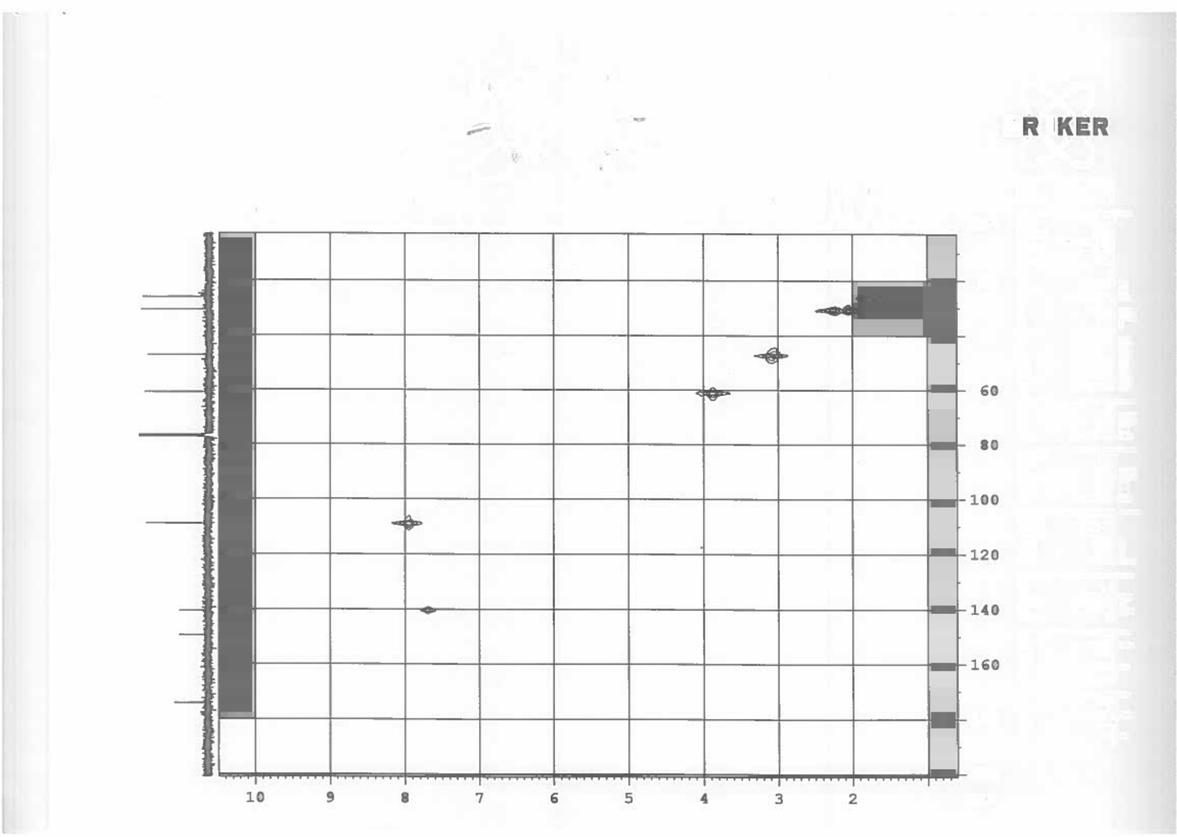

Figure S1. NMR spectra of organocatalyst **1**.

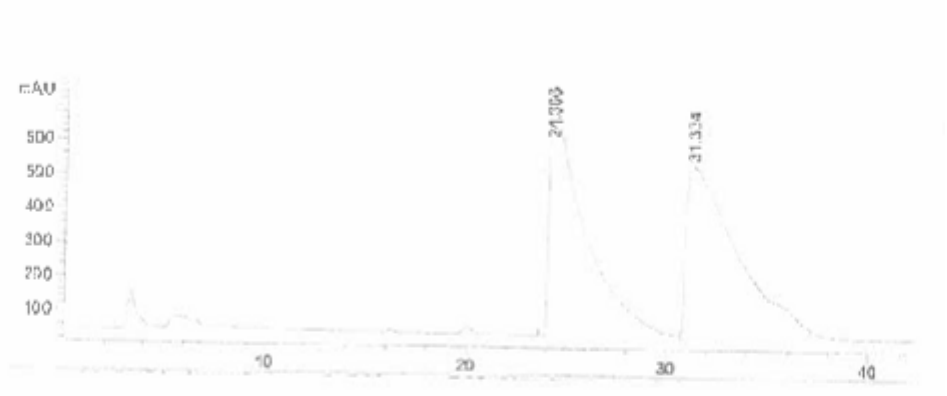

Figure S2. The reference GC chromatogram for the aldol reaction of acetone with 4-nitrobenzaldehyde. The retention times and intensities for *R*- and *S*-enantiomers are 24.3 and 31.3 min and 49 and 51, respectively.

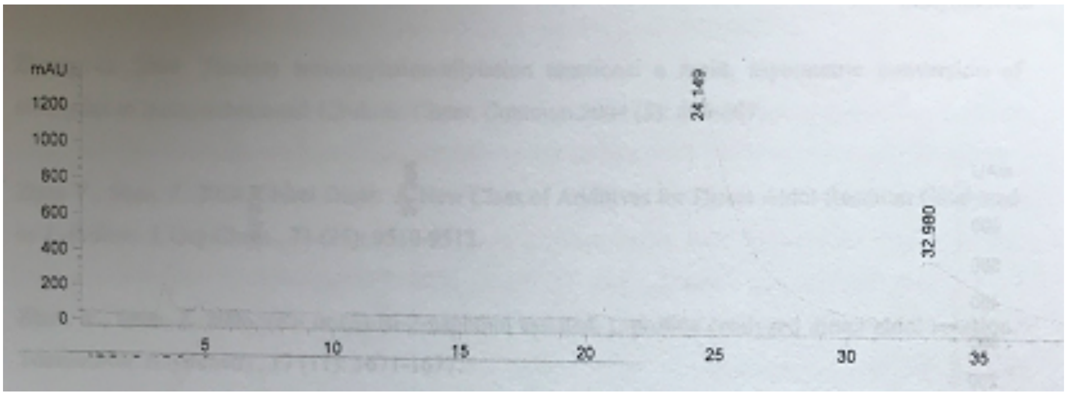

Figure S3. The GC chromatogram for the aldol reaction of acetone with 4-nitrobenzaldehyde catalyzed by **1** in the absence of additive. The retention times and intensities for *R*- and *S*-enantiomers are 24.1 and 32.0 min and 75 and 25, respectively.

Atomic coordinates for the TS structures in SYBYL format obtained by HF/6-31 + G(d)

@<TRIPOS>MOLECULE

***Re*-face catalyzed by 1**

72 74

SMALL

NO_CHARGES

@<TRIPOS>ATOM

1 C1 –0.9948 4.3938 0.9298 C

2 C2 –0.2404 3.2500 0.7215 C

3 C3 0.1923 2.9220 –0.5592 C

4 C4 –0.1511 3.7433 –1.6271 C

5 C5 –0.9106 4.8857 –1.4208 C

6 C6 –1.3303 5.2143 –0.1400 C

7 C7 0.9978 1.6756 –0.7926 C

8 O8 0.9302 0.7269 0.0576 O

9 H9 1.0266 1.4030 –1.8455 H

10 H10 –1.3225 4.6460 1.9226 H

11 H11 0.0096 2.6044 1.5429 H

12 H12 0.1712 3.4941 –2.6245 H

13 H13 –1.1724 5.5141 –2.2529 H

14 C14 –0.9969 –2.5644 –0.3670 C

15 C15 –0.9557 –3.9220 –0.6383 C

16 C16	0.2900	–4.5204	–0.7184	C
17 C17	1.4816	–3.8264	–0.5382	C
18 C18	1.3861	–2.4794	–0.2738	C
19 H19	–1.8591	–4.4724	–0.7769	H
20 H20	0.3376	–5.5740	–0.9248	H
21 H21	2.4347	–4.3035	–0.6020	H
22 N22	0.1643	–1.9013	–0.2075	N
23 N23	2.4074	–1.5918	–0.0471	N
24 H24	2.1068	–0.6410	0.1295	H
25 N25	–2.1157	–1.7910	–0.2337	N
26 H26	–2.0092	–0.8541	0.1039	H
27 C27	3.7510	–1.8285	–0.1756	C
28 C28	4.6561	–0.6633	0.2190	C
29 O29	4.2264	–2.8697	–0.5101	O
30 N30	4.0121	0.6594	0.2405	N
31 C31	5.1457	–0.8784	1.6712	C
32 H32	5.4886	–0.6730	–0.4697	H
33 C33	4.0246	1.2319	1.6038	C
34 C34	5.2079	0.5298	2.2562	C
35 H35	4.4292	–1.4784	2.2223	H
36 H36	6.0906	–1.4025	1.6859	H
37 H37	3.0893	1.0158	2.1050	H
38 H38	4.1513	2.3012	1.5519	H
39 H39	5.1425	0.5347	3.3367	H
40 H40	6.1321	1.0225	1.9728	H
41 C41	–3.4321	–2.1861	–0.4366	C

42	C42	–4.4586	–1.0792	–0.2410	C
43	O43	–3.7357	–3.2851	–0.7805	O
44	N44	–4.0046	0.0088	0.6026	N
45	C45	–4.7574	–0.4064	–1.5967	C
46	H46	–5.3496	–1.5707	0.1314	H
47	C47	–4.2722	1.3177	–0.0094	C
48	C48	–5.1887	0.9996	–1.1888	C
49	H49	–3.8560	–0.3591	–2.2020	H
50	H50	–5.5029	–0.9554	–2.1555	H
51	H51	–3.3508	1.7917	–0.3354	H
52	H52	–4.7414	1.9726	0.7127	H
53	H53	–5.0980	1.7169	–1.9953	H
54	H54	–6.2240	0.9920	–0.8631	H
55	C55	3.6261	1.3291	–0.8340	C
56	C56	2.8169	2.4743	–0.7557	C
57	H57	2.8265	3.1086	–1.6243	H
58	H58	2.7668	3.0068	0.1739	H
59	C59	3.9551	0.7630	–2.1913	C
60	H60	3.5431	–0.2298	–2.3313	H
61	H61	3.5647	1.4036	–2.9677	H
62	H62	5.0307	0.6951	–2.3162	H
63	H63	0.1626	–0.8878	–0.0780	H
64	H64	–1.9152	6.1014	0.0240	H
65	C65	–3.8926	–0.1087	1.9983	C
66	C66	–3.4003	0.8749	2.7485	C
67	H67	–3.3513	0.7700	3.8154	H

68 H68 –3.0548 1.8029 2.3328 H

69 C69 –4.3599 –1.4020 2.6234 C

70 H70 –3.8355 –2.2684 2.2323 H

71 H71 –4.1907 –1.3742 3.6907 H

72 H72 –5.4204 –1.5595 2.4556 H

@<TRIPOS>BOND

1 1 2 2

2 1 6 Ar

3 1 10 1

4 2 3 Ar

5 2 11 1

6 3 4 Ar

7 3 7 1

8 4 5 Ar

9 4 12 1

10 5 6 Ar

11 5 13 1

12 6 64 1

13 7 8 2

14 7 9 1

15 14 15 2

16 14 22 Ar

17 14 25 Ar

18 15 16 2

19 15 19 1

20 16 17 Ar

21 16 20 1

22 17 18 2

23 17 21 1

24 18 22 Ar

25 18 23 Ar

26 22 63 1

27 23 24 1

28 23 27 Ar

29 25 26 1

30 25 41 1

31 27 28 1

32 27 29 2

33 28 30 1

34 28 31 1

35 28 32 1

36 30 33 1

37 30 55 Ar

38 31 34 1

39 31 35 1

40 31 36 1

41 33 34 1

42 33 37 1

43 33 38 1

44 34 39 1

45 34 40 1

46 41 42 1

47 41 43 2

48 42 44 1

49 42 45 1

50 42 46 1

51 44 47 1

52 44 65 1

53 45 48 1

54 45 49 1

55 45 50 1

56 47 48 1

57 47 51 1

58 47 52 1

59 48 53 1

60 48 54 1

61 55 56 Ar

62 55 59 1

63 56 57 1

64 56 58 1

65 59 60 1

66 59 61 1

67 59 62 1

68 65 66 2

69 65 69 1

70 66 67 1

71 66 68 1

72 69 70 1

73 69 71 1

74 69 72 1

@<TRIPOS>MOLECULE

***Si*-face catalyzed by 1**

72 74

SMALL

NO_CHARGES

@<TRIPOS>ATOM

1 C1 –1.1596 –2.1653 –0.8134 C

2 C2 –1.1816 –3.3346 –1.5568 C

3 H3 –2.1103 –3.7872 –1.8235 H

4 C4 0.0360 –3.8769 –1.9297 C

5 H5 0.0352 –4.7836 –2.5066 H

6 C6 1.2600 –3.3074 –1.5929 C

7 H7 2.1909 –3.7379 –1.8898 H

8 C8 1.2266 –2.1497 –0.8505 C

9 N9 0.0310 –1.6242 –0.4952 N

10 H10 0.0663 –0.7464 0.0243 H

11 N11 –2.2392 –1.4669 –0.3524 N

12 H12 –2.0752 –0.6660 0.2259 H

13 C13 –3.5795 –1.7678 –0.5555 C

14 O14 –3.9475 –2.7217 –1.1663 O

15 C15 –4.5486 –0.7417 0.0157 C

16 H16	–5.4196	–1.3044	0.3298	H
17 C17	–4.9396	0.2609	–1.0905	C
18 H18	–4.0943	0.4467	–1.7476	H
19 H19	–5.7533	–0.1151	–1.6954	H
20 C20	–5.2754	1.5237	–0.3023	C
21 H21	–5.2256	2.4210	–0.9069	H
22 H22	–6.2771	1.4522	0.1093	H
23 C23	–4.2463	1.5133	0.8256	C
24 H24	–3.3352	2.0311	0.5365	H
25 H25	–4.6185	1.9777	1.7289	H
26 N26	–3.9896	0.0875	1.0656	N
27 C27	–3.7660	–0.3820	2.3703	C
28 C28	–4.2306	–1.7835	2.6888	C
29 H29	–3.9707	–2.0323	3.7082	H
30 H30	–3.7770	–2.5301	2.0443	H
31 H31	–5.3071	–1.8735	2.5851	H
32 C32	–3.1739	0.3695	3.2963	C
33 H33	–2.8284	1.3677	3.1001	H
34 H34	–3.0390	–0.0039	4.2931	H
35 N35	2.2879	–1.4134	–0.3880	N
36 H36	2.0325	–0.6000	0.1559	H
37 C37	3.6170	–1.6307	–0.6282	C
38 O38	4.0460	–2.5140	–1.3055	O
39 C39	4.5846	–0.6521	0.0562	C
40 H40	5.2054	–0.2453	–0.7269	H
41 C41	5.4193	–1.3817	1.1177	C

42	H42	5.6759	–2.3853	0.8108	H
43	H43	6.3387	–0.8293	1.2773	H
44	C44	4.5270	–1.3149	2.3574	C
45	H45	3.7683	–2.0897	2.3230	H
46	H46	5.0793	–1.4416	3.2792	H
47	C47	3.8842	0.0706	2.2720	C
48	H48	2.8657	0.0886	2.6283	H
49	H49	4.4606	0.8099	2.8148	H
50	N50	3.9275	0.4097	0.8281	N
51	C51	3.5573	1.5863	0.3586	C
52	C52	3.8258	1.9110	–1.0860	C
53	H53	3.4764	1.1306	–1.7515	H
54	H54	3.3409	2.8350	–1.3582	H
55	H55	4.8948	2.0287	–1.2380	H
56	C56	2.8227	2.4951	1.1449	C
57	H57	2.8358	3.5124	0.7974	H
58	H58	2.9114	2.4032	2.2120	H
59	C59	0.9417	1.8951	0.8926	C
60	H60	0.6697	2.3178	1.8536	H
61	O61	0.8960	0.6235	0.7955	O
62	C62	0.4402	2.7443	–0.2463	C
63	C63	0.2715	2.2185	–1.5222	C
64	H64	0.5280	1.1930	–1.7142	H
65	C65	–0.2256	3.0064	–2.5508	C
66	H66	–0.3475	2.5891	–3.5346	H
67	C67	–0.5672	4.3297	–2.3118	C

68 H68 –0.9531 4.9404 –3.1079 H

69 C69 –0.4124 4.8604 –1.0381 C

70 H70 –0.6827 5.8826 –0.8433 H

71 C71 0.0863 4.0707 –0.0143 C

72 H72 0.1979 4.4900 0.9715 H

@<TRIPOS>BOND

1 1 2 2

2 1 9 Ar

3 1 11 Ar

4 2 3 1

5 2 4 2

6 4 5 1

7 4 6 Ar

8 6 7 1

9 6 8 2

10 8 9 Ar

11 8 35 Ar

12 9 10 1

13 11 12 1

14 11 13 1

15 13 14 2

16 13 15 1

17 15 16 1

18 15 17 1

19 15 26 1

20 17 18 1

21 17 19 1

22 17 20 1

23 20 21 1

24 20 22 1

25 20 23 1

26 23 24 1

27 23 25 1

28 23 26 1

29 26 27 1

30 27 28 1

31 27 32 2

32 28 29 1

33 28 30 1

34 28 31 1

35 32 33 1

36 32 34 1

37 35 36 1

38 35 37 Ar

39 37 38 2

40 37 39 1

41 39 40 1

42 39 41 1

43 39 50 1

44 41 42 1

45 41 43 1

46 41 44 1

47 44 45 1

48 44 46 1

49 44 47 1

50 47 48 1

51 47 49 1

52 47 50 1

53 50 51 2

54 51 52 1

55 51 56 Ar

56 52 53 1

57 52 54 1

58 52 55 1

59 56 57 1

60 56 58 1

61 59 60 1

62 59 61 2

63 59 62 1

64 62 63 Ar

65 62 71 Ar

66 63 64 1

67 63 65 Ar

68 65 66 1

69 65 67 Ar

70 67 68 1

71 67 69 Ar

72 69 70 1

73 69 71 2

74 71 72 1

@<TRIPOS>MOLECULE

***Re*-face catalyzed by 2**

78 80

SMALL

NO_CHARGES

@<TRIPOS>ATOM

1 C1 –0.2153 3.5767 –0.1521 C

2 C2 0.6848 2.8293 –0.9125 C

3 C3 2.0537 2.9233 –0.7147 C

4 C4 2.5369 3.7984 0.2586 C

5 C5 1.6553 4.5461 1.0179 C

6 C6 0.2834 4.4323 0.8181 C

7 H7 3.5975 3.8879 0.4138 H

8 H8 2.0312 5.2220 1.7659 H

9 H9 –0.3933 5.0173 1.4164 H

10 N10 3.4894 0.9057 –0.8226 N

11 H11 2.8506 0.1224 –0.7655 H

12 N12 –2.1596 2.0954 –0.2576 N

13 H13 –1.5415 1.4325 0.1517 H

14 C14 –3.4298 1.7356 –0.5367 C

15	C15	–3.7335	0.2406	–0.3798	C
16	O16	–4.2467	2.5193	–0.9457	O
17	N17	–5.0537	–0.0030	0.1438	N
18	C18	–3.7551	–0.4439	–1.7588	C
19	H19	–2.9846	–0.2169	0.2585	H
20	C20	–5.9727	–0.5353	–0.8549	C
21	C21	–5.2237	–0.3521	–2.1736	C
22	H22	–3.0782	0.0186	–2.4674	H
23	H23	–3.4542	–1.4792	–1.6387	H
24	H24	–6.9146	–0.0013	–0.8530	H
25	H25	–6.1835	–1.5882	–0.6720	H
26	H26	–5.4356	0.6267	–2.5847	H
27	H27	–5.4997	–1.1012	–2.9077	H
28	C28	4.6567	0.9009	–0.1840	C
29	C29	5.0865	–0.3810	0.5460	C
30	O30	5.4321	1.8299	–0.1436	O
31	N31	4.0642	–1.4329	0.6393	N
32	C32	6.2281	–1.0805	–0.2026	C
33	H33	5.3997	–0.0679	1.5300	H
34	C34	4.3032	–2.5131	–0.3463	C
35	C35	5.4929	–2.0136	–1.1642	C
36	H36	6.8801	–0.3685	–0.6877	H
37	H37	6.8183	–1.6547	0.5051	H
38	H38	3.4179	–2.6690	–0.9378	H
39	H39	4.5454	–3.4221	0.1945	H
40	H40	5.1457	–1.4660	–2.0329	H

41	H41	6.1107	–2.8324	–1.5126	H
42	H42	0.3078	2.1595	–1.6668	H
43	C43	–1.7047	3.4687	–0.3971	C
44	H44	–2.2429	4.1065	0.2939	H
45	H45	–1.9499	3.8100	–1.3956	H
46	C46	3.0090	2.0792	–1.5367	C
47	H47	2.5095	1.7296	–2.4307	H
48	H48	3.8661	2.6645	–1.8348	H
49	C49	–5.3478	0.1389	1.4806	C
50	C50	–4.4759	0.5640	2.4046	C
51	H51	–4.7842	0.6560	3.4284	H
52	H52	–3.4695	0.8590	2.1782	H
53	C53	–6.7628	–0.2057	1.8790	C
54	H54	–7.4729	0.4556	1.3934	H
55	H55	–7.0172	–1.2242	1.6041	H
56	H56	–6.8834	–0.1035	2.9487	H
57	C57	3.0990	–1.4654	1.5193	C
58	C58	2.0542	–2.4256	1.4505	C
59	H59	2.2754	–3.3509	0.9498	H
60	H60	1.5206	–2.5671	2.3758	H
61	C61	3.0021	–0.3602	2.5377	C
62	H62	2.1048	–0.4764	3.1273	H
63	H63	3.8555	–0.3927	3.2076	H
64	H64	2.9804	0.6140	2.0646	H
65	C65	0.8650	–1.5608	0.2634	C
66	O66	1.4976	–1.1309	–0.7514	O

67 C67 –0.1628 –2.6589 0.0574 C

68 C68 –1.1191 –2.9421 1.0267 C

69 C69 –0.1779 –3.3757 –1.1332 C

70 C70 –2.0652 –3.9355 0.8211 C

71 H71 –1.1344 –2.3811 1.9467 H

72 C72 –1.1201 –4.3732 –1.3404 C

73 H73 0.5402 –3.1300 –1.8928 H

74 C74 –2.0645 –4.6587 –0.3636 C

75 H75 –2.8020 –4.1384 1.5783 H

76 H76 –1.1234 –4.9215 –2.2663 H

77 H77 –2.7967 –5.4297 –0.5267 H

78 H78 0.4801 –0.8165 0.9685 H

@<TRIPOS>BOND

1 1 2 Ar

2 1 6 Ar

3 1 43 1

4 2 3 Ar

5 2 42 1

6 3 4 Ar

7 3 46 1

8 4 5 2

9 4 7 1

10 5 6 Ar

11 5 8 1

12 6 9 1

13 10 11 1

14 10 28 Ar

15 10 46 1

16 12 13 1

17 12 14 Ar

18 12 43 1

19 14 15 1

20 14 16 2

21 15 17 1

22 15 18 1

23 15 19 1

24 17 20 1

25 17 49 Ar

26 18 21 1

27 18 22 1

28 18 23 1

29 20 21 1

30 20 24 1

31 20 25 1

32 21 26 1

33 21 27 1

34 28 29 1

35 28 30 2

36 29 31 1

37 29 32 1

38 29 33 1

39 31 34 1

40 31 57 2

41 32 35 1

42 32 36 1

43 32 37 1

44 34 35 1

45 34 38 1

46 34 39 1

47 35 40 1

48 35 41 1

49 43 44 1

50 43 45 1

51 46 47 1

52 46 48 1

53 49 50 2

54 49 53 1

55 50 51 1

56 50 52 1

57 53 54 1

58 53 55 1

59 53 56 1

60 57 58 Ar

61 57 61 1

62 58 59 1

63 58 60 1

64 61 62 1

65 61 63 1

66 61 64 1

67 65 66 2

68 65 67 1

69 65 78 1

70 67 68 Ar

71 67 69 Ar

72 68 70 Ar

73 68 71 1

74 69 72 Ar

75 69 73 1

76 70 74 Ar

77 70 75 1

78 72 74 Ar

79 72 76 1

80 74 77 1

@<TRIPOS>MOLECULE

***Si*-face catalyzed by 2**

78 80

SMALL

NO_CHARGES

@<TRIPOS>ATOM

1 C1 –0.2078 –2.8792 –1.9946 C

2 C2 0.8015 –1.9263 –2.0321 C

3	C3	2.1390	–2.2757	–1.9229	C
4	C4	2.4761	–3.6220	–1.8409	C
5	C5	1.4818	–4.5891	–1.8489	C
6	C6	0.1446	–4.2201	–1.9092	C
7	H7	3.5091	–3.9158	–1.7666	H
8	H8	1.7470	–5.6301	–1.7910	H
9	H9	–0.6202	–4.9768	–1.8841	H
10	N10	3.1122	–0.5318	–0.5300	N
11	H11	2.1966	–0.2875	–0.1888	H
12	N12	–1.9410	–1.5769	–0.8687	N
13	H13	–1.2329	–0.9389	–0.5599	H
14	C14	–3.1696	–1.5083	–0.3246	C
15	C15	–3.2769	–0.5954	0.8999	C
16	O16	–4.1092	–2.1566	–0.7128	O
17	N17	–4.5687	0.0523	1.0496	N
18	C18	–3.1378	–1.4683	2.1768	C
19	H19	–2.4890	0.1472	0.8571	H
20	C20	–5.3660	–0.5996	2.0669	C
21	C21	–4.3130	–1.0557	3.0691	C
22	H22	–3.2217	–2.5169	1.9175	H
23	H23	–2.1736	–1.3268	2.6509	H
24	H24	–5.9288	–1.4411	1.6651	H
25	H25	–6.0670	0.1054	2.4966	H
26	H26	–4.6553	–1.8662	3.7032	H
27	H27	–4.0359	–0.2226	3.7081	H
28	C28	4.1894	0.0446	0.0040	C

29 C29	4.0758	0.6767	1.4011	C
30 O30	5.2827	0.0621	–0.5104	O
31 N31	2.8047	0.4897	2.1260	N
32 C32	5.0704	–0.0101	2.3448	C
33 H33	4.2838	1.7296	1.2941	H
34 C34	2.8628	–0.7327	2.9590	C
35 C35	4.2949	–1.2496	2.7899	C
36 H36	6.0043	–0.2290	1.8511	H
37 H37	5.2668	0.6419	3.1908	H
38 H38	2.1137	–1.4397	2.6290	H
39 H39	2.6659	–0.4772	3.9910	H
40 H40	4.3358	–2.0150	2.0238	H
41 H41	4.6718	–1.6812	3.7090	H
42 H42	0.5390	–0.8858	–2.0949	H
43 C43	–1.6528	–2.4317	–2.0102	C
44 H44	–1.8688	–1.9014	–2.9342	H
45 H45	–2.3189	–3.2795	–1.9663	H
46 C46	3.1847	–1.1871	–1.8325	C
47 H47	4.1789	–1.5913	–1.9443	H
48 H48	3.0432	–0.4578	–2.6246	H
49 C49	–5.1593	0.8137	0.0501	C
50 C50	–6.4770	0.9859	–0.0673	C
51 H51	–6.8679	1.6461	–0.8182	H
52 H52	–7.1921	0.4790	0.5516	H
53 C53	–4.2050	1.5266	–0.8776	C
54 H54	–4.7512	2.1915	–1.5333	H

55 H55 –3.4849 2.1154 –0.3177 H

56 H56 –3.6519 0.8331 –1.5014 H

57 C57 1.8585 1.3923 2.2376 C

58 C58 1.6880 2.4749 1.3399 C

59 H59 1.2485 3.3546 1.7805 H

60 H60 2.4952 2.6966 0.6650 H

61 C61 0.8021 1.1829 3.2890 C

62 H62 0.0861 1.9899 3.2672 H

63 H63 0.2781 0.2506 3.1129 H

64 H64 1.2450 1.1456 4.2781 H

65 C65 0.2597 1.7953 0.2443 C

66 O66 0.4352 0.5660 –0.0240 O

67 C67 0.2917 2.8164 –0.8730 C

68 C68 0.9475 2.5430 –2.0674 C

69 C69 –0.3604 4.0358 –0.7284 C

70 C70 0.9615 3.4766 –3.0926 C

71 H71 1.4391 1.5969 –2.1899 H

72 C72 –0.3519 4.9714 –1.7526 C

73 H73 –0.8870 4.2564 0.1855 H

74 C74 0.3127 4.6943 –2.9383 C

75 H75 1.4726 3.2530 –4.0124 H

76 H76 –0.8669 5.9077 –1.6282 H

77 H77 0.3197 5.4160 –3.7357 H

78 H78 –0.5382 2.0386 0.9490 H

@<TRIPOS>BOND

1 1 2 Ar

2 1 6 Ar

3 1 43 1

4 2 3 Ar

5 2 42 1

6 3 4 Ar

7 3 46 1

8 4 5 Ar

9 4 7 1

10 5 6 Ar

11 5 8 1

12 6 9 1

13 10 11 1

14 10 28 Ar

15 10 46 1

16 12 13 1

17 12 14 Ar

18 12 43 1

19 14 15 1

20 14 16 2

21 15 17 1

22 15 18 1

23 15 19 1

24 17 20 1

25 17 49 1

26 18 21 1

27 18 22 1

28 18 23 1

29 20 21 1

30 20 24 1

31 20 25 1

32 21 26 1

33 21 27 1

34 28 29 1

35 28 30 2

36 29 31 1

37 29 32 1

38 29 33 1

39 31 34 1

40 31 57 2

41 32 35 1

42 32 36 1

43 32 37 1

44 34 35 1

45 34 38 1

46 34 39 1

47 35 40 1

48 35 41 1

49 43 44 1

50 43 45 1

51 46 47 1

52 46 48 1

53 49 50 2

54 49 53 1

55 50 51 1

56 50 52 1

57 53 54 1

58 53 55 1

59 53 56 1

60 57 58 Ar

61 57 61 1

62 58 59 1

63 58 60 1

64 61 62 1

65 61 63 1

66 61 64 1

67 65 66 2

68 65 67 1

69 65 78 1

70 67 68 Ar

71 67 69 Ar

72 68 70 Ar

73 68 71 1

74 69 72 Ar

75 69 73 1

76 70 74 Ar

77 70 75 1

78 72 74 Ar

79 72 76 1

80 74 77 1

@<TRIPOS>MOLECULE

***Re*-face catalyzed by 3**

78 80

SMALL

NO_CHARGES

@<TRIPOS>ATOM

1 C1 –1.2171 –2.2823 –0.8733 C

2 C2 0.2188 –2.4528 –1.3952 C

3 C3 0.4373 –3.8374 –2.0150 C

4 C4 –0.5716 –4.1026 –3.1341 C

5 C5 –2.0031 –3.9601 –2.6165 C

6 C6 –2.2232 –2.5810 –1.9943 C

7 H7 1.4535 –3.8921 –2.3970 H

8 H8 0.3582 –1.7184 –2.1844 H

9 H9 –1.3816 –2.9732 –0.0541 H

10 H10 –0.4120 –3.4046 –3.9555 H

11 H11 –0.4097 –5.0988 –3.5370 H

12 H12 –2.7156 –4.1161 –3.4220 H

13 H13 –2.1985 –4.7323 –1.8744 H

14 H14 –2.1182 –1.8165 –2.7634 H

15 H15 –3.2258 –2.4955 –1.5948 H

16 H16	0.3464	–4.6006	–1.2549	H
17 N17	1.2251	–2.0862	–0.3972	N
18 H18	1.3799	–1.0935	–0.3410	H
19 N19	–1.3913	–0.9265	–0.3643	N
20 H20	–0.7103	–0.2323	–0.5991	H
21 C21	–2.4262	–0.5715	0.4174	C
22 C22	–2.4526	0.9020	0.8494	C
23 O23	–3.2763	–1.3468	0.7859	O
24 N24	–3.7609	1.5117	0.7306	N
25 C25	–2.1813	1.0261	2.3558	C
26 H26	–1.7197	1.4559	0.2794	H
27 C27	–4.5561	1.3258	1.9334	C
28 C28	–3.5613	0.8412	2.9998	C
29 H29	–1.4511	0.3092	2.7127	H
30 H30	–1.7978	2.0224	2.5517	H
31 H31	–5.3428	0.5951	1.7759	H
32 H32	–5.0237	2.2688	2.2026	H
33 H33	–3.7350	–0.2031	3.2197	H
34 H34	–3.6527	1.3994	3.9252	H
35 C35	1.7247	–2.8575	0.5699	C
36 C36	2.7722	–2.2154	1.5064	C
37 O37	1.4797	–4.0240	0.7597	O
38 N38	2.9277	–0.7582	1.3736	N
39 C39	2.3734	–2.3769	2.9746	C
40 H40	3.7047	–2.7170	1.2931	H
41 C41	2.1617	–0.0452	2.4233	C

42 C42	1.4776	–1.1625	3.2121	C
43 H43	1.8873	–3.3243	3.1505	H
44 H44	3.2625	–2.3222	3.5959	H
45 H45	1.4693	0.6427	1.9689	H
46 H46	2.8628	0.5055	3.0411	H
47 H47	0.4854	–1.3506	2.8178	H
48 H48	1.3743	–0.9071	4.2596	H
49 C49	3.6995	–0.1460	0.5123	C
50 C50	3.6571	1.2602	0.3342	C
51 H51	4.5280	1.6775	–0.1432	H
52 H52	3.3053	1.8503	1.1605	H
53 C53	4.5534	–0.9676	–0.4184	C
54 H54	5.3235	–1.4833	0.1473	H
55 H55	3.9684	–1.7085	–0.9478	H
56 H56	5.0381	–0.3267	–1.1400	H
57 C57	–4.3638	1.8359	–0.4718	C
58 C58	–5.6760	2.0542	–0.6021	C
59 H59	–6.0765	2.3609	–1.5498	H
60 H60	–6.3732	1.9444	0.2055	H
61 C61	–3.4411	2.0081	–1.6552	C
62 H62	–2.6782	2.7534	–1.4512	H
63 H63	–2.9384	1.0819	–1.9115	H
64 H64	–4.0062	2.3299	–2.5195	H
65 C65	2.2243	1.4335	–0.9306	C
66 O66	1.2183	0.7679	–0.5293	O
67 C67	2.1233	2.9425	–0.9976	C

68 C68 1.1488 3.6135 –0.2686 C

69 C69 2.9803 3.6754 –1.8104 C

70 C70 1.0427 4.9943 –0.3412 C

71 H71 0.4711 3.0457 0.3407 H

72 C72 2.8762 5.0567 –1.8873 C

73 H73 3.7294 3.1669 –2.3948 H

74 C74 1.9073 5.7207 –1.1490 C

75 H75 0.2818 5.5028 0.2244 H

76 H76 3.5426 5.6104 –2.5249 H

77 H77 1.8212 6.7911 –1.2088 H

78 H78 2.7634 1.0510 –1.8001 H

@<TRIPOS>BOND

1 1 2 1

2 1 6 1

3 1 9 1

4 1 19 1

5 2 3 1

6 2 8 1

7 2 17 1

8 3 4 1

9 3 7 1

10 3 16 1

11 4 5 1

12 4 10 1

13 4 11 1

14 5 6 1

15 5 12 1

16 5 13 1

17 6 14 1

18 6 15 1

19 17 18 1

20 17 35 Ar

21 19 20 1

22 19 21 Ar

23 21 22 1

24 21 23 2

25 22 24 1

26 22 25 1

27 22 26 1

28 24 27 1

29 24 57 1

30 25 28 1

31 25 29 1

32 25 30 1

33 27 28 1

34 27 31 1

35 27 32 1

36 28 33 1

37 28 34 1

38 35 36 1

39 35 37 2

40 36 38 1

41 36 39 1

42 36 40 1

43 38 41 1

44 38 49 2

45 39 42 1

46 39 43 1

47 39 44 1

48 41 42 1

49 41 45 1

50 41 46 1

51 42 47 1

52 42 48 1

53 49 50 Ar

54 49 53 1

55 50 51 1

56 50 52 1

57 53 54 1

58 53 55 1

59 53 56 1

60 57 58 2

61 57 61 1

62 58 59 1

63 58 60 1

64 61 62 1

65 61 63 1

66 61 64 1

67 65 66 2

68 65 67 1

69 65 78 1

70 67 68 Ar

71 67 69 Ar

72 68 70 Ar

73 68 71 1

74 69 72 Ar

75 69 73 1

76 70 74 Ar

77 70 75 1

78 72 74 Ar

79 72 76 1

80 74 77 1

@<TRIPOS>MOLECULE

***Si*-face catalyzed by 3**

78 80

SMALL

NO_CHARGES

@<TRIPOS>ATOM

1 C1 –1.5246 1.4585 1.5009 C

2 C2 –0.0915 1.7048 2.0018 C

3 C3 –0.0223 2.9136 2.9418 C

4 C4 –0.9832 2.7547 4.1221 C

5 C5 –2.4113 2.5073 3.6352 C

6 C6 –2.4707 1.2992 2.6992 C

7 H7 1.0001 3.0173 3.2951 H

8 H8 0.2024 0.8278 2.5715 H

9 H9 –1.8465 2.3076 0.9097 H

10 H10 –0.6656 1.9209 4.7476 H

11 H11 –0.9419 3.6437 4.7462 H

12 H12 –3.0769 2.3497 4.4797 H

13 H13 –2.7750 3.3908 3.1133 H

14 H14 –2.1900 0.3999 3.2465 H

15 H15 –3.4778 1.1485 2.3330 H

16 H16 –0.2631 3.8148 2.3925 H

17 N17 0.8976 1.7693 0.9267 N

18 H18 1.2893 0.8755 0.6867 H

19 N19 –1.5440 0.2741 0.6495 N

20 H20 –0.7188 –0.2894 0.5882 H

21 C21 –2.5881 –0.0424 –0.1321 C

22 C22 –2.4304 –1.3249 –0.9632 C

23 O23 –3.5937 0.6256 –0.2081 O

24 N24 –3.5271 –2.2577 –0.7841 N

25 C25 –2.5293 –1.0127 –2.4627 C

26 H26 –1.4879 –1.7977 –0.7246 H

27 C27 –4.6369 –1.9445 –1.6695 C

28 C28 –4.0355 –1.0449 –2.7627 C

29 H29 –2.0733 –0.0645 –2.7256 H

30 H30	–2.0159	–1.7990	–3.0074	H
31 H31	–5.4324	–1.4284	–1.1417	H
32 H32	–5.0432	–2.8685	–2.0694	H
33 H33	–4.4538	–0.0506	–2.6961	H
34 H34	–4.2356	–1.4284	–3.7574	H
35 C35	0.9867	2.7370	0.0117	C
36 C36	2.0441	2.4924	–1.0943	C
37 O37	0.3522	3.7632	0.0098	O
38 N38	1.5931	1.4682	–2.0543	N
39 C39	2.1967	3.7151	–1.9979	C
40 H40	2.9715	2.1914	–0.6371	H
41 C41	0.5878	2.1173	–2.9203	C
42 C42	1.0309	3.5939	–2.9957	C
43 H43	2.1709	4.6380	–1.4391	H
44 H44	3.1503	3.6451	–2.5114	H
45 H45	–0.3847	2.0184	–2.4563	H
46 H46	0.5518	1.6449	–3.8866	H
47 H47	0.2150	4.2440	–2.7166	H
48 H48	1.3412	3.8537	–4.0009	H
49 C49	2.1710	0.3136	–2.2933	C
50 C50	3.1466	–0.3002	–1.4676	C
51 H51	3.8168	–0.9446	–2.0115	H
52 H52	3.6497	0.3072	–0.7374	H
53 C53	1.6961	–0.4823	–3.4851	C
54 H54	0.6254	–0.6408	–3.4475	H
55 H55	1.9325	0.0349	–4.4091	H

56 H56 2.1852 –1.4441 –3.5048 H

57 C57 –3.7321 –2.9980 0.3698 C

58 C58 –4.9236 –3.4833 0.7296 C

59 H59 –5.0072 –4.1113 1.5964 H

60 H60 –5.8271 –3.2850 0.1866 H

61 C61 –2.4998 –3.3139 1.1841 C

62 H62 –2.7561 –3.9793 1.9977 H

63 H63 –1.7444 –3.7983 0.5725 H

64 H64 –2.0561 –2.4216 1.6104 H

65 C65 2.1435 –1.5458 –0.3558 C

66 O66 1.2219 –0.9238 0.2428 O

67 C67 3.2862 –2.1253 0.4504 C

68 C68 4.0949 –3.1246 –0.0799 C

69 C69 3.5158 –1.6924 1.7505 C

70 C70 5.1264 –3.6731 0.6668 C

71 H71 3.9155 –3.4844 –1.0798 H

72 C72 4.5493 –2.2360 2.4995 C

73 H73 2.8724 –0.9439 2.1732 H

74 C74 5.3590 –3.2255 1.9599 C

75 H75 5.7407 –4.4492 0.2455 H

76 H76 4.7168 –1.8948 3.5059 H

77 H77 6.1566 –3.6501 2.5433 H

78 H78 1.8547 –2.2241 –1.1602 H

@<TRIPOS>BOND

1 1 2 1

2 1 6 1

3 1 9 1

4 1 19 1

5 2 3 1

6 2 8 1

7 2 17 1

8 3 4 1

9 3 7 1

10 3 16 1

11 4 5 1

12 4 10 1

13 4 11 1

14 5 6 1

15 5 12 1

16 5 13 1

17 6 14 1

18 6 15 1

19 17 18 1

20 17 35 Ar

21 19 20 1

22 19 21 Ar

23 21 22 1

24 21 23 2

25 22 24 1

26 22 25 1

27 22 26 1

28 24 27 1

29 24 57 1

30 25 28 1

31 25 29 1

32 25 30 1

33 27 28 1

34 27 31 1

35 27 32 1

36 28 33 1

37 28 34 1

38 35 36 1

39 35 37 2

40 36 38 1

41 36 39 1

42 36 40 1

43 38 41 1

44 38 49 2

45 39 42 1

46 39 43 1

47 39 44 1

48 41 42 1

49 41 45 1

50 41 46 1

51 42 47 1

52 42 48 1

53 49 50 Ar

54 49 53 1

55 50 51 1

56 50 52 1

57 53 54 1

58 53 55 1

59 53 56 1

60 57 58 2

61 57 61 1

62 58 59 1

63 58 60 1

64 61 62 1

65 61 63 1

66 61 64 1

67 65 66 2

68 65 67 1

69 65 78 1

70 67 68 Ar

71 67 69 Ar

72 68 70 Ar

73 68 71 1

74 69 72 Ar

75 69 73 1

76 70 74 Ar

77 70 75 1

78 72 74 Ar

79 72 76 1

80 74 77 1
